# Antimicrobial peptide and sequence variation along a latitudinal gradient in two anurans

**DOI:** 10.1186/s12863-020-00839-1

**Published:** 2020-03-30

**Authors:** Maria Cortázar-Chinarro, Yvonne Meyer-Lucht, Tom Van der Valk, Alex Richter-Boix, Anssi Laurila, Jacob Höglund

**Affiliations:** 1 Animal Ecology/Department of Ecology and Genetics, Uppsala University, Norbyvägen 18D, SE-75236 Uppsala, Sweden; 2 Centre for Paleogenetics Svante Arrhenius väg 20C, SE-106 91 Stockholm, Sweden

**Keywords:** Rana, MHC, AMPs, Trans-specific polymorphism, Multi-locus system

## Abstract

**Background:**

While there is evidence of both purifying and balancing selection in immune defense genes, large-scale genetic diversity in antimicrobial peptides (AMPs), an important part of the innate immune system released from dermal glands in the skin, has remained uninvestigated. Here we describe genetic diversity at three AMP loci (Temporin, Brevinin and Palustrin) in two ranid frogs (*Rana arvalis* and *R. temporaria*) along a 2000 km latitudinal gradient. We amplified and sequenced part of the Acidic Propiece domain and the hypervariable Mature Peptide domain (~ 150-200 bp) in the three genes using Illumina Miseq and expected to find decreased AMP genetic variation towards the northern distribution limit of the species similarly to studies on MHC genetic patterns.

**Results:**

We found multiple loci for each AMP and relatively high gene diversity, but no clear pattern of geographic genetic structure along the latitudinal gradient. We found evidence of trans-specific polymorphism in the two species, indicating a common evolutionary origin of the alleles. Temporin and Brevinin did not form monophyletic clades suggesting that they belong to the same gene family. By implementing codon evolution models we found evidence of strong positive selection acting on the Mature Peptide. We also found evidence of diversifying selection as indicated by divergent allele frequencies among populations and high Theta k values.

**Conclusion:**

Our results suggest that AMPs are an important source of adaptive diversity, minimizing the chance of microorganisms developing resistance to individual peptides.

## Background

Vertebrates fight pathogens using both the adaptive and the innate immune systems. The adaptive immune system is composed of highly specialized tissues and systemic cells, which synthetize antibodies and recognize an infinite diversity of antigens [1]. The adaptive immune system usually clears the infection and protects the host against reinfection with the same pathogen [1, 2]. The innate immune system provides a non-specific response to pathogens, and when a pathogen is detected it acts immediately with an early induced inflammatory response recognized by non-specific effectors [2]. The vertebrate innate immune system includes macrophages and neutrophils [3], natural killer cells [4] and antimicrobial peptides (AMPs [5, 6]), the latter often being secreted from the granular glands on the dermal layer of the skin. AMPs are generally short (15–45 amino acid residues) [7], cationic [8], amphipathic [9] and α helical [10]. These molecules are found in a great variety of taxa such as mollusks [11], fish [12], amphibians [13], birds [14] and mammals, including humans [15].

**Fig. 1:**
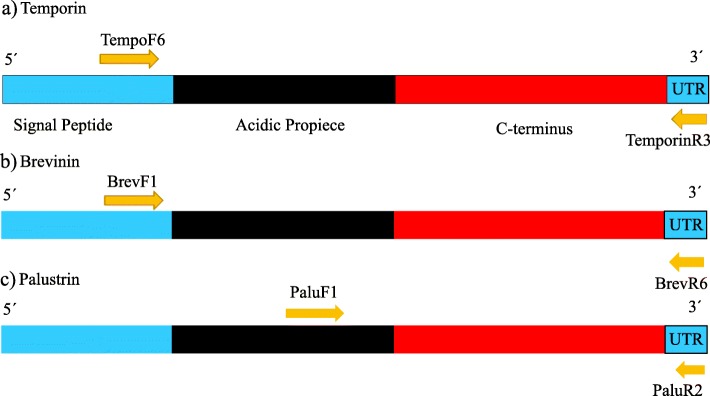
Primer design and location of primers within the genes

In amphibians, AMPs consist of a highly conserved Signal Peptide, an Acidic Propiece domain and a hypervariable C-terminus domain [16], Fig. 1). Amphibian AMPs exhibit potent activity against antibiotic resistant bacteria, protozoa, yeast, virus and fungi by killing cells via disrupting the membrane integrity [17, 18]. They have been implicated as a defense against the lethal fungus *Batrachochytrium dendrobatidis (Bd)* causing global amphibian population declines (e.g., [19–21]). It has been firmly established that AMPs may be encoded by duplicated genes [17, 22]. A high number of amphibian AMPs has been characterized with the aim to develop peptide-based therapeutic agents [23, 24]. However, most of the AMP studies have focused on the antimicrobial peptide variation between amphibian species, and the genetic variation at the population level has received less consideration. Indeed, while there is evidence of geographic variation in effectiveness of antimicrobial defenses in various organism groups [22, 25–29], population-level variation in peptide profiles [27, 29] and positive selection acting on the Mature Peptide domain [6, 7, 16], little attention has been directed towards understanding the evolutionary forces acting on the genes coding for AMPs [17, 22, 30, 31]. This is especially the case for the role of selection acting upon sequence variation in AMP genes, and how this variation is distributed within populations as well as at larger geographical scales.

Intraspecific genetic diversity is a fundamental dimension of biodiversity. Intraspecific genetic diversity is a reflection of both past and current evolutionary processes, as well as an indicator of a population’s potential for adaptation to future stressors [32]. In temperate and boreal regions, historical colonization events such as postglacial colonization have a strong effect on genetic variation at all spatial scales. Genetic diversity both within and between species generally decreases with increasing latitude [33]. Moreover, understanding the role of selection and gene flow is pivotal for understanding the genetic diversity displayed in contemporary populations. For instance, diversifying selection in different parts of the gradient, especially in cases in which a cline coincides with a demographic shift, may cause population divergence [34]. Thus, genetic diversity patterns could be attributed to diversifying selection, different demography and/or gene flow along a geographical gradient [35, 36]. To which extent demography, selection, gene flow and their interactions contribute to shape large-scale genetic variation remains a complex and unresolved issue.

In this study, we assessed variation in AMP genes in two ranid frogs, the moor frog *Rana arvalis* and the common frog *R. temporaria*, along a latitudinal gradient from northern Germany to the northern margin of the distribution of the two species. We investigated both the historical post-glacial colonization patterns along the latitudinal gradient across northern Europe (Additional File 3: Figure 1) and current evolutionary bottlenecks (selective forces, drift/demography, migration) driving AMP intraspecific genetic variation. *R. temporaria* shows an almost unidirectional postglacial colonization of Scandinavia, colonizing most of Sweden from the north via Finland and meeting another lineage colonizing from the south via Denmark in southernmost Sweden [37, 38]. *R. arvalis* shows a different pattern of dual postglacial colonization from south and north to Scandinavia, the contact zone being situated in central northern Sweden [39, 40]. Populations at the northern edge are smaller, more fragmented and isolated relative to populations at the core of the distribution range [41–43]. As a consequence of decreasing effective population size at range margins, many species show lower genetic variation at the edge of their distribution range in concordance with the central-marginal hypothesis [44]. *R. arvalis* shows a clear and structured allele distribution with a decreasing pattern of genetic variation towards northern latitudes at microsatellite loci as well as at the major histocompatibility complex (MHC) class II exon 2, an important component of the adaptive immune response [40]. These results are in concordance with the central-marginal hypothesis. Following previous results, we expected the lowest genetic diversity at the AMP loci at the northern edge of the distribution of both species.

To our knowledge, this is the first investigation of large-scale genetic variation in AMPs 1) within and between populations of a species and 2) comparing two different species. We compared AMP genetic variation among 14 *R. arvalis* and 17 *R. temporaria* populations from northern Germany to northern Scandinavia. We expected a decrease in AMP genetic variation towards the northern distribution limit of the species. We characterized skin antimicrobial peptides genetically using ultra-deep Illumina sequencing in order to elucidate the underlining evolutionary processes acting on AMP genes. Specifically, we asked: 1) are patterns of AMP genetic variation influenced by demographic history? 2) is there evidence of selection at AMP loci? and 3) to which extent is selection affecting AMP gene characteristics?

## Results

### AMP diversity: Miseq run summary

The total number of reads obtained by run varied from 2.597.554 to 5.124.580. We obtained an average of 4.048.930 reads ±987.541 (s.d.) with intact primer barcode information from four separate runs (Additional File 1: Table 1). The average number of reads per amplicon was 218.503,14 ± 35.720 (s.d.). Amplicons with < 300 reads were discarded. We included replicated individuals for each of the four Miseq runs (15–20%; (Additional File 1: Table 1). Replicates were randomly assigned across different pools to avoid false allele identification. All replicates produced the same alleles in each sample. We extracted DNA from a total of 320 individuals (150 *R. arvalis* and 170 *R,temporaria*). For all three genes (Temporing, Brevinin and Palustrin), we amplified and sequenced a total of 849 amphibian DNA samples, plus the duplicates (Raar = 376; Rate = 473; See Table 1; (Additional File 1: Table 1). For Temporin, we genotyped (275) (Raar = 109; Rate = 166), for Brevinin 296 (Raar = 131; Rate = 165) and for Palustrin 278 (Raar = 136; Rate = 142) individuals. Among the 849 sequenced samples, we assigned 58 valid AMP sequences with length variation within and among loci (from 111 to 204 bp) by using the DOC method [45].

**Table 1:**
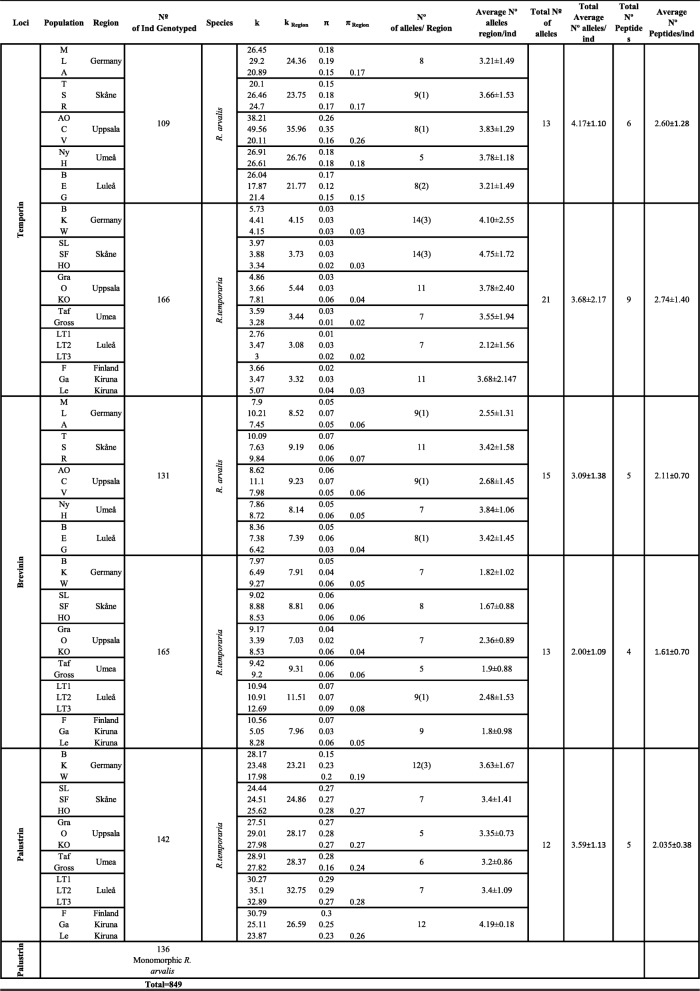
Diversity statistics summary for Population/Region of Temporin, Brevinin and Palustrin group of genes in two different frog species (*R. arvalis* and *R. temporaria*). K; Theta K pairwise nucleotide comparison. K_Region_; Theta K pairwise comparation within regions. π; nucleotide diversity and π_Region_; nucleotide diversity within regions. Number of alleles in brackets represent unique alleles within regions

### Multi-locus family genes

We detected more than two alleles per individual in both species, indicating multiple copies in all sequenced AMP genes. Number of valid AMP alleles per individual varied between one and 11 in Temporin, one and seven in Brevinin, and one and six in Palustrin. The average number of gene copies per AMP ranged from 2 to 4 (Temporin; Raar = 4.17 ± 1.10[s.d.], Rate = 3.68 ± 2.17 [s.d.]; Brevinin; Raar = 3.09 ± 1.38[s.d.], Rate = 2.00 ± 1.09 [s.d.] and Palutrin; Rate = 3.59 ± 1.13[s.d.]).

### AMP nucleotide gene diversity

Allele frequency pie-charts showed a non-structured genetic pattern from northern to southern populations at all loci for both species (Additional File 4: Figure 2). Pairwise nucleotide differences (Theta *k*) varied considerably among species and group of genes. However, Theta *k* values were very similar within regions, showing the same allelic pattern in the allele frequency plots throughout the gradient (Table 1; (Additional File 4: Figure 2; Additional File 5: Figure 3).

In Temporin, three out of 21 alleles (Rate_Temp*21, Rate_Temp*20, Rate_Temp*17) were widespread at high frequencies in all *R. temporaria* populations (Additional File 4: Figure 2; Additional File 5: Figure 3). Similarly, the three most common alleles (Raar_Rate_Temp*02, Raar_Rate_Temp*05 and Raar_Temp*17) out of the 13 in *R. arvalis* were found in all populations at high frequency (Additional File 4: Figure 2; Additional File 5: Figure 3). In Palustrin, Rate_Palu*07 and Raar_Rate_Palu*04 were the most frequent alleles out of the 12 alleles in *R. temporaria* (Additional File 4: Figure 2; Additional File 5: Figure 3) and found in all populations. Palustrin was monomorphic in *R. arvalis*. In Brevinin the most predominant allele Raar_Rate_Brev*02 was found in all populations along the gradient in both species (Additional File 4: Figure 2; Additional File 5: Figure 3). In *R. arvalis,* four (Raar_Brev*10, 11, 12, 16) Brevinin alleles out of the total of 14 were present in only southern populations from Uppsala to Germany.

**Fig. 2:**
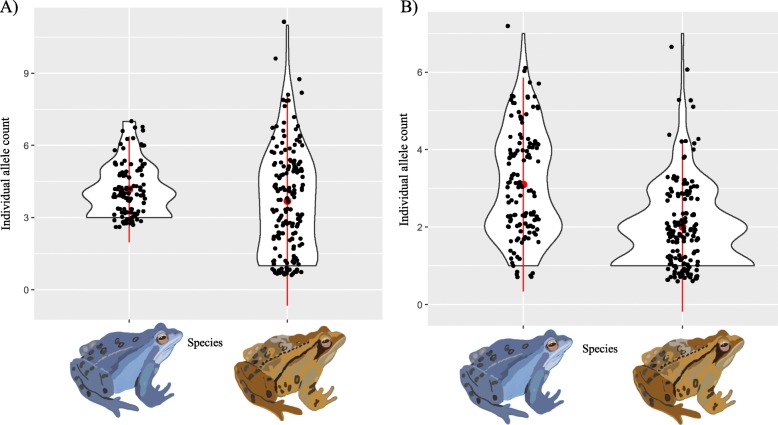
Number of alleles per individual (Individual allele count) observed in *R. arvalis* (blue) and *R.temporaria* (brown) in Temporin **a**) and in Brevinin **b**) group of genes. The Average number of alleles and the standard deviations are indicated in Red. To improve the visualization of Fig. 2, all values has been “jittered”. Frogs illustrations were created by A.Cortazar for this specific study

The number of alleles per region was significantly different among regions for the Brevinin group of genes in *R. arvalis* (F_4,9.62_ = 5.48, *P* = 0.014), but there was no difference in *R. temporaria* (F_5,25.78_ = 1.82, *P* = 0.14). The results were the opposite for the Temporin group of genes, where we found a higher number of alleles in the two southern regions in *R. temporaria* (F_5,10.85_ = 4.29, *P* = 0.021), but not in *R. arvalis* (F_4,8.53_ = 0.61, *P* = 0.41). For the Palustrin group of genes we found no significant differences in number of alleles among the regions in *R. temporaria* (F_5,10.78_ = 2.54, *P* = 0.092). The number of Brevinin alleles was significantly higher in *R. arvalis* than in *R. temporaria* (F_1, 25.34_ = 22.16, *p* < 0.001; Fig. 2), and almost so in Temporin (F_1,25.53_ = 5.36, *p* = 0.054).

### Phylogenetic analyses

**Fig. 3:**
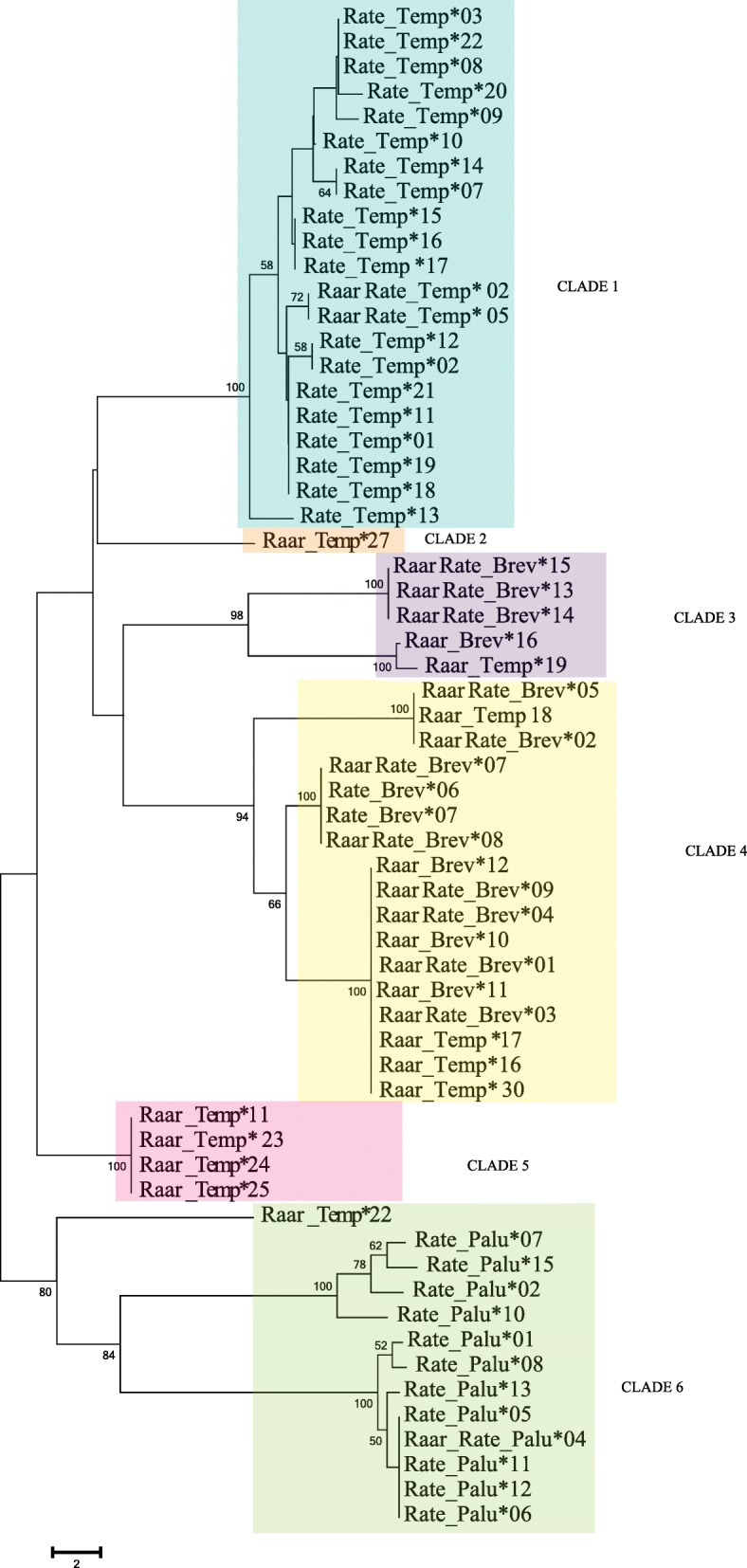
Molecular phylogram of nucleotide sequences of ranid an-timicrobial peptides reconstructed with neighbor joining methods for the three AMP genes. Bootstrap values from 1000 replicates greater than 50% are indicated on branches. Alleles that belong to the same group (Clade 1 to 6) are included in the same colored square. The valid alleles were named following the nomenclature by Klein (1975) for MHC loci: a four-digit abbreviation of the species name followed by species_gene*numeration, e.g. Raar_Brev*01

The Neighbor-joining (NJ) phylogenetic tree and the haplotype network generated for Temporin showed that *R. arvalis* and *R. temporaria* alleles were distinctly separated with the exception of two alleles which were present in both species (Raar_Rate_Temporin*02,05; Fig. 3; (Additional File 6: Figure 4). In contrast, the trees generated for Brevinin and Palustrin indicated that many alleles (species-specific and shared) were not separated between the two species. The alleles of both species were clustered into three, five and two clades for Temporin, Brevinin and Palustrin, respectively (Additional File 7: Figure 5).

We constructed a NJ phylogenetic tree where all alleles earlier assigned to Temporin, Brevinin and Palustrin were clustered in seven different groups/clades according to the connected branches and nodes of the tree (Fig. 3). Surprisingly, Temporin and Brevinin alleles were widespread and did not form monophyletic clades in the phylogenetic trees, occurring in the same groups/clades. Consequently, we consider Temporin and Brevinin as a part of the same gene family and they were grouped together for consecutive analyses.

### AMP peptide diversity

**Fig. 4:**
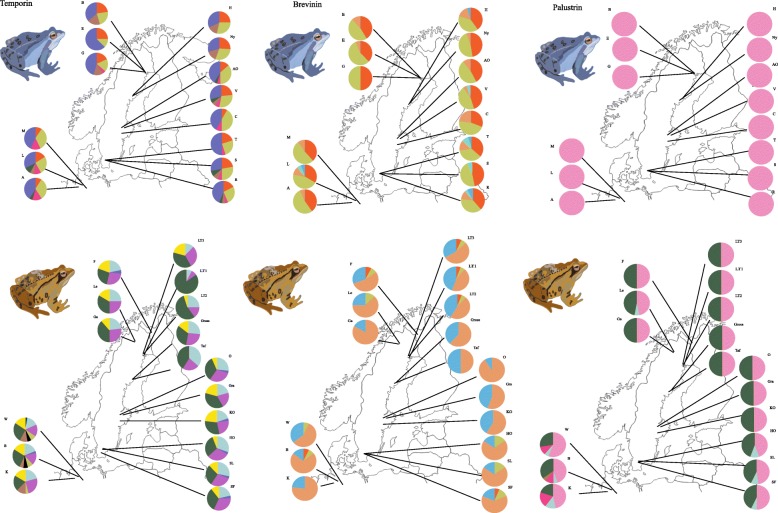
Peptide allele frequency pie charts Temporin, Brevinin and Palustrin group of genes 14 *R. arvalis* populations (upper row: A: Altwarmbüchen; M: Mardof; Se: Seebeckwiesen; S: Sjöhusen; T: Tvedöra; R: Räften; AÖ: Österbybruk; V: Valsbrunna; C: Crayfish/Almby; H: Holmsjön; Ny: Nydalasjön; B: Besbyn; E: Ernäs; G: Gemträsket) and 17 R.temporaria populations (lower row: B: Altwarmbüchen; K: Schneeren – Kuhteich; W: Osterloh – Wienhausen; HO: Höör; SF: Sjöbo S; SL: Östra Odarslöv; Grä: Gränby; KO: Kolvia; Ö: Österbybruk; Taf: Tafteå; Gross: Grossjön; LT1: Besbyn; LT2: Mockträsket; LT3: Gemträsket; Ga: Gällivare; Le: Leipojärvi; F: Kilpisjärvi). Colour coding scheme for the alleles is given in the (Additional file 10: Figure S8). Frogs illustrations were created by A.Cortazar for this specific study

When DNA sequences were translated to amino-acids, variant frequency showed a structured pattern from northern to southern populations in Temporin for *R. temporaria* and in Brevinin for *R. arvalis* (Temporin; F_5,10.85_ = 4.29, *p* = 0.021, Brevinin; F_4,8.51_ = 3.59, *p* = 0.054; Fig. 4; (Additional File 8: Figure 6 and Additional File 9: Figure 7), indicating lower peptide variation at higher latitudes. The Palustrin group did not show any pattern of peptide variation along the gradient in *R. temporaria* (Fig. 4; (Additional file 10: Figure S8, Additional file 11: Figure S9, Additional file 12: Figure 10, Additional file 13: Figure S11 and Additional file 14: Figure S12). Palu_Amino*05 was the only amino-acid variant found in *R. arvalis* for Palustrin (Fig. 4; From (Additional file 10: Figure S8, Additional file 11: Figure S9, Additional file 12: Figure 10, Additional file 13: Figure S11 and Additional file 14: Figure S12). The Temporin variant Temp_Amino*07, and two amino-acid variants Temp_Brev_Amino*01,02 present at both Brevinin and Temporin, were the only functional amino-acid variants shared between the two species (Fig. 4; (Additional File 8: Figure 6 and Additional File 9: Figure 7).

### Adaptive evolution/ positive selection in AMPs multi-locus gene family

We examined patterns of nucleotide diversity (π) and average pairwise nucleotide differences (Theta *k*) within each of the 46 Mature Peptide and Acidic Propiece domains among four set of clades (Temporin-Brevinin) and 12 Mature Peptide and Acidic Propiece domains among two set of clades (Palustrin) (Table 2). The diversity at the Mature Peptide domain was higher (π_Mature Peptide_ = 0.437; π_Mature Peptide_ = 0.35, k_Mature Peptide_ = 14.439; k_Mature Peptide_ = 23.136) and more divergent than at the Acidic Propiece domains (π_Acidic Propiece_ = 0.115; π_Acidic Propiece=_0.116; k_Acidic Propiece_ = 11.76; k_Acidic Propiece_ = 2.78) in both Temporin-Brevinin and Palustrin group of genes, respectively. The net charges of the Mature Peptide and the Acidic Propiece showed a negative relationship among Temporin-Brevinin and Palustrin sequences. The corresponding “lm” slopes were − 2.75 ± 0.45 for Temporin-Brevinin and − 1.02 ± 0.41 for Palustrin group of genes, indicating coordinated changes between the Mature Peptide domain and the Acidic Propiece domain. Net charges of the Mature Peptide and the Acidic Propiece domains for Temporin-Brevinin and Palustrin ranged from − 6 to 3 and from − 1 to 4, respectively (Additional File 15: Figure 13).

**Table 2:**
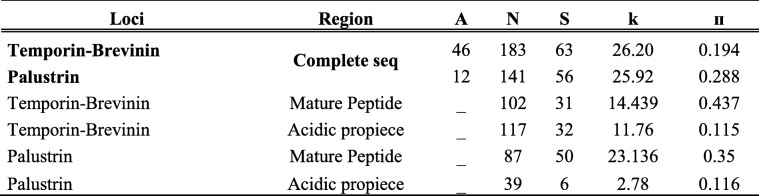
Diversity statistics for Temporin-Brevinin and Palustrin. The diversity estimates were calculated based on the complete sequence, the Acidic Propiece and the Mature Peptide. A; the number of alleles, N; number of base pairs (bp), S; number of segregating sites, k; pairwise nucleotide differences, π; nucleotide diversity index

We found evidence of positive selection on specific codon sites in the Mature Peptide and the Acidic Propiece domains using the maximum likelihood models implemented in CODEML [46]. The model allowing for selection fitted the data significantly better than neutral models only for the Temporin-Brevinin group (Additional File 2; Table 2, Additional File 16: Figure 14 and Additional File 16), while the evidence of selection on specific codon sites in the Palustrin group was not significantly better for the selection model compared to the neutral model. The models allowing for selection (PAML, FEL and REL) identified a total number of 14 sites under selection in Temporin-Brevinin and five in Palustrin. The Mature Peptide domain displayed 12 out of 14 and five out of five, for Temporin-Brevinin and Palustrin respectively, of the positively selected sites within the hypervariable region. Ten sites (3, 21, 40, 43, 44, 47, 49, 50, 51, 56) out of 14 in Temporin-Brevinin group were identified by at least two different approaches with posterior probabilities > 0.95 (See Aditional File 2).

## Discussion

We studied variation in AMP genes in two amphibians along a latitudinal gradient from northern Germany to the northern margin of distribution of the two species (~ 2000 km). We investigated the relative contribution of selective forces, drift/demography and migration driving AMP genetic variation. We expected that genetic variation at the AMP loci would be lower towards the species distribution margin in the north [40]. Contrary to our expectations, we found genetic variation in all AMPs was largely equally distributed among the regions. The Theta *k* values did not differ systematically among populations and regions, suggesting similar effects of selection and demography shaping the genetic variation at AMP loci. However, we found evidence for reduced latitudinal variation at the peptide level in Brevinin for *R. arvalis*, and in Temporin for *R. temporaria*. Moreover, we found trans-specific polymorphism between *R. arvalis* and *R. temporaria*, alleles in the Temporin and Brevinin group of genes clustering to a large extent by allele similarity. We also detected signatures of historical positive selection on AMP genes, as well as multiple lines of evidence suggesting strong diversifying selection maintaining adaptive variation within the Mature Peptide region.

### Neutral vs. functional diversity?

As a consequence of decreasing effective population size (N_e_) at range margins, many widely distributed species show lower genetic variation in populations at the edge of their distribution range [41, 42]. While *R. arvalis* and *R. temporaria* have slightly different postglacial recolonization history in Scandinavia, both species have colonized Scandinavian Peninsula both from the south (via Denmark) and the north (via Finland) and lost neutral genetic variability during this process [37, 40, 42]. Nevertheless, a study of Scandinavian *R. arvalis* found a clear and structured allelic distribution with a decreasing pattern of genetic variation towards northern latitudes at microsatellite loci as well as at the major histocompatibility complex (MHC) class II exon 2, an important component of the adaptive immune response [40]. These results were mainly explained by a complex pattern of varying levels of drift and selection along the latitudinal gradient which is influenced by colonization history of the species [40, 47]. Both in *R. temporaria* and *R. arvalis* the highest genetic variation both in neutral and adaptive allele frequencies is found in the southernmost regions of the latitudinal gradient [43, 48, 49].

We found that AMP alleles are largely equally distributed within species in *R. arvalis* and *R. temporaria* populations. Opposed to the findings on MHC, this study presents no clear loss of alleles towards the north of the gradient. However, we found a decrease in functional amino-acid diversity towards the northern latitudes in Brevinin in *R. arvalis* and in Temporin in *R. temporaria*. This may suggest 1) independent effects of ecological and/or demographic conditions on genetic and amino-acid variability of AMPs, 2) differential importance of pathogens along the gradient 3) and/or drift. Furthermore, the differences in AMP diversity and allele/peptide geographic distribution along the gradient between the species might indicate different selective pressures acting on AMP genes in the two species.

In general, the intensity of species interactions decreases towards higher latitudes [50, 51]. The lower AMP amino-acid variability might be associated with the weaker biotic interactions at higher latitudes, which are strongly dependent on environmental conditions. Gurnier and coworkers [52] suggested that stochastic changes in environmental temperature, humidity and precipitation may affect parasitic and infectious microorganism diversity disproportionally, weakening the effect of pathogens at northern latitudes. Infectious diseases are also likely to be fewer in the north [50] and hence select for fewer and functionally similar AMP alleles at northern latitudes, while southern populations may have to cope with a more diverse flora of pathogens. Also anthropogenic alteration of natural environments and its effect on the intensity of species interactions can be weaker in the north [53]. We hypothesize that the latitudinal variation in biotic and abiotic interactions may contribute to the observed functional pattern found at amino-acid level. The lower genetic variation commonly observed at high latitudes [40, 54, 55] might suggest lower adaptive potential of AMPs (Temporin in *R. temporaria* and Brevinin in *R. arvalis*) against climate mediated changes in parasite and disease regimes in northern populations.

Our study highlights the need for additional work on the genetic basis for disease resistance in amphibians facing population declines in many parts of the world due to emerging infectious diseases [21, 56–58]. *Bd* infections are widespread in southern and central Sweden [59, 60], but data from northern Scandinavia are largely lacking [60, 61]. Several studies have investigated the complex relationship between skin microbiota composition and AMPs as a direct measure to fight *Bd* infections (e.g. [62, 63]), while other studies have shown that purified AMPs inhibit *Bd* growth under laboratory conditions [64, 65]. Therefore, understanding how genetic variation in AMPs is distributed across large spatial scales is important for future studies and may help to explain direct effects of AMPs on disease resistance and *Bd*-related declines.

### Copy number and trans-species polymorphism variation

Other vertebrates have evolved multiple copies per individuals in AMPs [66] and specifically in some amphibian species [31]. However, this is the first study to show that AMP group of genes in *R.arvalis* and *R.temporaria* is a clear multi-locus system by using NGS techniques with multiple alleles per individual as a result of gene duplication. A minimum of four loci were found in Temporin and three in Brevinin and Palustrin. The importance of duplicated genes as a reservoir of raw genetic material and its contribution in evolution has been recognized for long [67], but very little is known about AMPs copy number variation and their location within amphibian genomes. Earlier studies have shown the presence of more than one MHC class II loci in several species [68–70], but before our study this was not described for AMPs along a latitudinal gradient in ranids.

One of our main results is that the alleles within each AMP clade/paralog were clustered by allele and not by species, and similar alleles occurred in both species. This pattern is a hallmark of trans-specific polymorphism (TSP), which has been found especially in immune genes in a variety of taxa [71–73]. In our study, topologies for phylogenetic trees based on nucleotide sequences constructed for Brevinin, Temporin and Palustrin group of genes show that alleles share more similarity between, rather than within, species. This suggests that these AMPs have originated before the two species diverged at least 3.0 mya [74] or more than 10 mya [75]. Therefore, incomplete lineage sorting or hybridization is an unlikely explanation for the observed TSP. Our results are in concordance with Duda et al. [6] who showed that all alleles in Temporin, Ranalexin and Gaegurin loci clustered together within the ranid phylogeny, strengthening the idea of AMPs originating via concerted evolution before the divergence of the species.

Temporin alleles in *R. arvalis* (Raar_Temp*23,24,11,25,27,22,19,18,16,17,30) were grouped in two distinct clades, supporting a recent genetic duplication of Raar_Temp*23,24,11,25 *in R. arvalis* after the two species diverged. Rest of the alleles (Raar_Temp*27,22,19,18,16,17,30) awere more similar to Brevinin than Temporin alleles. The similarity between Temporin and Brevinin group of genes can be explained by several duplication events followed by subsequent episodes of evolutionary divergence. To demonstrate TSP conclusively, further research is needed to elucidate the causes of similarity between the groups of loci and the locations of these genes within the genomes.

### Selection on AMPs

Our results indicate that the AMPs in *R. temporaria* and *R. arvalis* exhibit the same characteristics as observed in other amphibians [6, 16, 17], including high levels of polymorphism, differences in sequence length and strong signatures of selection within the Mature Peptide region (C-terminal antimicrobial-coding region). We also found that the three antimicrobial peptides have evolved in a coordinated manner as a result of a maintained charge balance between the Acidic Propiece and the Mature Peptide over evolutionary time, which is in concordance with studies on mammalian defensins [76]. Interestingly, this was not the case in an earlier study [6], which suggested AMPs in hylids, but not in ranids, evolved in a coordinated manner. We hypothesize that under the coordinated evolution scenario, evolutionary processes might be driven by selective forces derived from the composition of the pathogen communities to which the species and populations are exposed. However, no data exist to test this hypothesis. Therefore, further investigations are needed regarding pathogen community composition in specific environments and how the bacterial communities drive coevolution in AMPs.

We found footprints of historical positive selection on specific amino-acid positions within both the Acidic Propiece and the Mature Peptide. The majority of the codons under positive selection are situated within the Mature Peptide, suggesting a strong selective pressure on the hypervariable Mature Peptide region, and more relaxed selection within the Acidic Propiece. Pathogen-driven selection is a well-known mechanism in MHC genes (e.g. [77–80]) but how these mechanisms act on AMP genes is not clear. Following the same principle as for MHC genes, a potential explanation for a strong selective pressure within the Mature Peptide domain in AMP genes may be due to differences in pathogen species composition along the latitudinal gradient. Moreover, temporal variation in pathogen-driven selection could also play a role on the Mature Peptide domain. In general, our results confirm the hypothesis that the hypervariable Mature Peptide domain is a result of adaptation to challenges by pathogens leading to increasing genetic diversity [6, 16]. Signatures of selection found this study could be contrasted with other markers such as neutral markers. However, given the complexity of multi-locus systems, such analyses of selection should be interpreted with caution.

## Conclusions

We investigated genetic diversity as well as selective forces shaping AMP diversity in two species of amphibians over a latitudinal gradient. We described novel AMP sequences and high AMP genetic variation in both species. We found strong evidence for trans-specific polymorphism and the estimated number of loci is high for each AMP. Our data support positive selection within the Mature Peptide domain. The evolution and diversification of this extensive family of hypervariable genes can be due to the contribution of different processes such as speciation events, gene duplication and targeted hyper-mutations, and we suggest the gene complex has ultimately evolved under diversifying selection. From a conservation perspective, identifying and characterizing AMPs is important for ultimately understanding the adaptive processes and the ability of populations to combat novel or altered pathogens. Hence, our results will facilitate further studies on the evolutionary and conservation ecology in amphibians.

## Methods

### Sample collection and DNA extraction

We sampled *R. arvalis* eggs in five regions, from northern Germany (Hanover) to northern Sweden (Luleå, Table 3). The eggs were collected at three sites in each region, with the exception of one region (Umeå), where we collected at two sites. For *R. temporaria*, we took samples in seven regions from northern Germany (Hanover) to northernmost Finland (Kilpisjärvi). We collected the eggs at three sites in each region except the regions in northern central Sweden (Umeå), northernmost Sweden (Kiruna) and northern Finland (Kilpisjärvi), where we collected eggs at two, two and one site, respectively. The eggs were collected in spring 2014 and 2015 except in Kilpisjärvi where the eggs were collected in 2009. The average distance between collection sites within a region was 20 km (range 8 to 50 km) for both species (Table 3). At each site we collected ca. ten eggs from ten freshly laid clutches. The species coexist in many of our sampling locations and the species were identified in the field by differences in the color of the jelly surrounding the eggs [81]. The eggs were transported to the laboratory in Uppsala and kept at 16 °C. After hatching, the tadpoles (stage 25, [82]) were euthanized with an overdose of MS222, preserved in 96% ethanol and stored at 4 °C until DNA extraction.

**Table 3:**
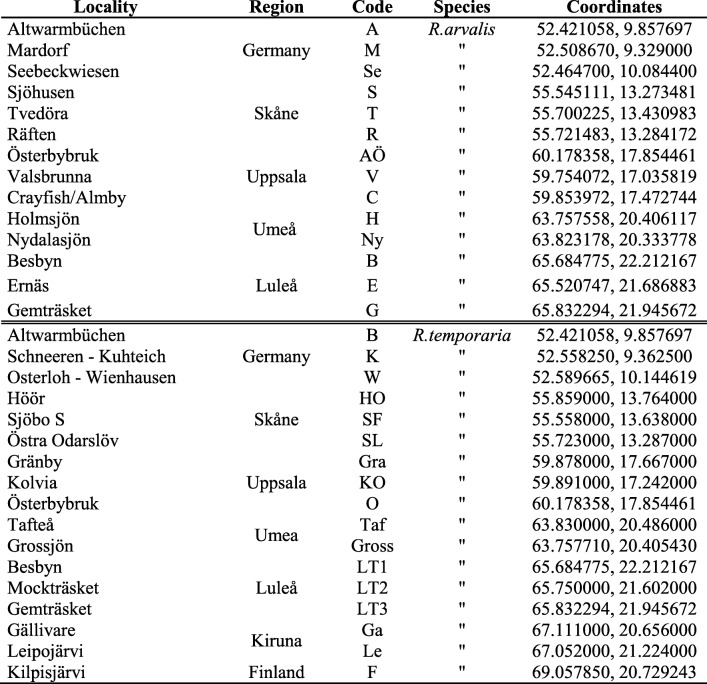
Summary of the sampling populations: locality name, Region, code, species and the geographic coordinates (Lat, Long) are shown for each population

Genomic DNA was extracted from ten individuals per site (one individual/clutch). We extracted genomic DNA from a total of 320 individuals (150 *R. arvalis* and 170 *R. temporaria*), using the DNeasy Blood and Tissue kit (Qiagen® Sollentuna, Sweden). Purity and concentration of DNA were determined with a NanoDrop® 2000 spectrophotometer and Qubit®3.0 fluorometer Quantitation Kit (Invitrogen™). Species verification was carried out by mtDNA cytochrome b amplification followed by the addition of *HaeIII* restriction enzyme (Palo and Merilä 2003). Digestion by *HaeIII* produces different, easily distinguishable banding patterns in *R. arvalis* and *R. temporaria*.

### Primer design, antimicrobial peptide amplification and preparation for sequencing

We used the primers designed by [17] for the amplification of Brevinin loci (see Table 4). PCR products were visualized and isolated from agarose gels (1.5%). The targets bands were excised from the gel and extracted using the MinElute Gel Extraction Kit (Qiagen® Sollentuna, Sweden) as Brevinin primers produced multiple PCR bands in *R. arvalis* and *R. temporaria*. We sequenced the resultant PCR product from the 3’end and 5’end and used it to design the primer pair BrevMF1 (5′-TTCAAGTTTGTGGCATCCCG-3′) and BrevR6 (5′- CAAGTTTCCAAAGTTCAACAT-3′). We designed primers for Temporin loci (summarized in Table 4) using several available *R. temporaria*, *R. japonica* and *R. versabilis* temporin cDNA sequences (Accession number: Y09394.1, Y09393.1, Y09395.1 [83];, Accession: AB593694.1 [84];; Accession: AM113510.1 [85];. PCR products were visualized and isolated from agarose gels (1.5%). The target bands were excised from the gel and extracted using the MinElute Gel Extraction Kit (Qiagen® Sollentuna, Sweden). The resulting PCR product was partially sequenced from the 3′ and used to design the primer pair TempoF6 (5′- GTCCCAGTGAAATACAGTTTTTGTA − 3′) and TemporinR3 (Table 4). Finally, we designed primers for Palustrin loci: PaluF1, PaluR2 (summarized in Table 4) using available Palustrin cDNA *R. versabilis* sequences (Accession: AM113507.1, AM745092.1 [86];. PCR products were visualized and isolated from agarose gels (1.5%). The resulting product was sequenced for DNA-sequence and fragment length verification. All PCR products were standard Sanger-sequenced at Macrogen Europe (Netherlands).

**Table 4:**
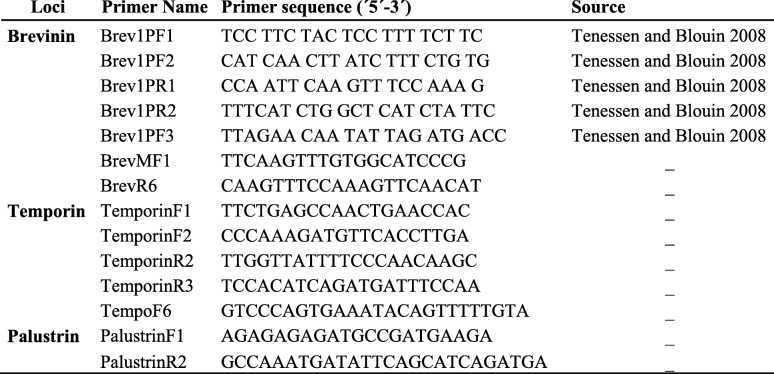
Summary of primers used in the study. Primers with ‘- ‘were designed for this study

The forward primers were positioned within the “Signal Peptide” (Brevinin, Temporin) or in the “Acidic Propiece” (Palustrin) region, the most conserved regions of the antimicrobial peptides, and all the reverse primers were positioned in the 3′ UTR-region (Fig. 1). The length of the fragments varied from 100 bp to 204 bp among loci and alleles within each locus. For Illumina Miseq sequencing, both forward and reverse primers for Temporin, Brevinin and Palustrin were modified with an individual 8 bp barcode and a sequence of three N (to facilitate cluster identification). Each amplicon was marked with an individual combination of a forward and a reverse barcode for identification. Reactions were conducted in a total volume of 20 μl containing 1 μl of genomic DNA, 2 μl of 10X Dream taq buffer (Thermo scientific lab), 0.4 μl of 2 mM of each dNTP, 0.5 μl of each 10 μM primer (BrevMF1-BrevR6; TempoF6-TempoR3; PaluF1-PaluR2, respectively), 1.5 μl of Bovine serum albumine (BSA; 5 mg/ml) and 0.25 μl of Dream taq DNA polymerase (5 U/μl, Thermo scientific lab) in deionized water. Thermocyling was performed on an ABI 2720 (Applied Biosystems®). All reactions were carried out using filter tips in separate (pre- and post-PCR) rooms, and negative controls were included in all amplifications to avoid contaminations. PCR products were run and visualized on a 1.5% agarose gel using gel green (BIOTIUM). To reduce the number of samples for subsequent purification, 3–9 PCR products with similar concentrations were pooled based on visual estimations from the gel image. These sample pools were run on 1.5% agarose gel, the target band was excised from the gel and extracted using the MinElute Gel Extraction Kit (Qiagen® Sollentuna, Sweden). The concentration of each sample pool was measured with Qubit 4 Fluorometer dsDNA assay kit (Invitrogen Life Technologies, Stockholm, Sweden). The final amplicon pooling was conducted according to the measured concentrations and consisted of equimolecular amounts of each sample. A total of eight final amplicon pools were generated per run, and libraries were prepared using the Illumina Truseq DNA PCR-Free Sample preparation kit (Illumina Inc., San Diego, CA). Eight pools each were combined into a Miseq run, and sequencing of four Miseq runs was carried out at the National Genomic Infrastructure (NGI), the SNP&SEQ Technology Platform hosted at SciLifeLab in Uppsala (Sweden).

### Miseq data analyses

Sequencing data were extracted from the raw data and paired-end reads were combined into single forward reads using FLASH [87], each of the eight amplicon pools was analyzed independently. In total, eight fastq files were generated per group of genes and transformed into fasta (multifasta) files using Avalanche NextGen package (DNA Baser Sequence Assembler v4 (2013), Heracle BioSoft, www.DnaBaser.com). The jMHC software [88] was used to remove primer sequences and unique tags, and to generate alignments of all alleles per amplicon. Generally, in multi-locus system studies using NGS techniques, rigorous quality control and filtering procedures have to be applied to distinguish PCR and sequencing artefacts from true alleles. In this particular case amplicon coverage and replication rate were remarkably high (ca. 20%). We assigned the most frequent alleles within each amplicon as valid AMP alleles that occurred in at least 3% of the reads [89, 90]. We discarded amplicons with < 300 reads from the analysis for quality reasons. In addition, we used the DOC method [91], not assuming any specific number of loci to identify and estimate the number of alleles (Ai) per individual. This procedure is based on the break point in sequencing coverage between alleles within each individual and avoids choosing a subjective threshold to separate true alleles from artefacts. Alleles are sorted top-down by coverage, followed by the calculation of the coverage break point (DOC statistic) around each allele. The allele with the highest DOC value is assumed to be the last true allele (see [91]). All valid alleles were imported and aligned by ClustalW in MEGA v7.0 [92]. Alleles were extensively compared to other sequences from the same putative locus in order to define the “Mature Peptide” and the “Acidic Propiece” region boundary within our alleles. Valid AMP alleles were named following the nomenclature suggested by [93] for MHC loci: a four-digit abbreviation of the species name followed by gene species*numeration, e.g. Raar_Brev*01.

### Data analyses

Relative allele frequencies were estimated for each AMP (Brevinin, Temporin and Palustrin) with ARLEQUIN v. 3.5 [94].. Allele frequency plots were created in R using the “ggplot2” package (Wickham 2016). We calculated the number of pairwise nucleotide differences by population (Theta *k*) and within region (Theta k_Region_) with DNAsp v.5 (Librado and Rozas 2009). We tested for differences in number of loci between the populations, regions and species by running a Generalized Mixed Model (GLMM) in R using the package ‘lme4’ [95]. Allele frequency was considered as the response variable, region and species as fixed factors and (population: region) as random factors of the model.

We constructed two phylogenetic trees to illustrate the phylogenetic relationship among AMPs sequences from different gene families (Temporin, Brevinin and Palustrin group of genes): 1) separately by genes, 2) all genes together, present in both species. We used the Neighbor method with bootstrapping (1000 replicates) implemented in MEGA v7.0 [92]. We also constructed a haplotype network per gene and per gene and species by using Minimum Spanning network inference [96] in the software PopART.

Relative amino-acid frequencies were estimated for each AMP with ARLEQUIN v. 3.5 (Excoffier, et al. 2010). Allele frequency plots were created in R using the “ggplot2” package [97]. AMP nucleotide sequences were grouped according to the phylogenetic tree based on amino-acid sequences. Some nucleotide sequences differed only by synonymous substitutions and translated into the same amino acid sequence (e.g Rate_Temp*06, Rate_Temp*22 and Rate_Temp*08 were named as Temp_Amino*01; see the phylogenetic tree based on Amino-acid sequences; (Additional File 8: Figure 6).

Nucleotide diversity (π), number of segregating sites (S), average number of pairwise nucleotide differences (Theta *k*) and Tajima’s D (D) were calculated for the entire AMP region, for the Acidic Propiece and for the Mature Peptide for every locus in DNAsp v.5 [98]. Net charges of the Acidic Propiece and the Mature Peptide were compared to determine whether the total charge of these domains showed a negative relationship which by definition means that both domains evolve in a coordinate manner. Net charges were calculated at PepCalc.com – Peptide property calculator platform. Signatures of natural selection at specific codon sites were also detected by using the common method on the ratios of non-synonymous to synonymous nucleotide substitution (dN/dS or ω) using the method CODEML in the package PAML [46]. We compared the two model pairs M2-M1 and M8-M7, the model pairs were tested with a log-likelihood test (LTR). We used the Bayes empirical Bayes (BEB) approach to identify significantly positive selected codon sites from the model M2 and M8. The tree files for PAML were constructed using a maximum likelihood approach in MEGA v7.0 [92]. We also used the random effect likelihood (REL) and the effect likelihood (FEL) with (HyPhy) package [99] implemented in the datamonkey server [100] to detect codons subjected to positive selection to contrast with the results obtained by CODEML. Average dN/dS (ω) was estimated with SLAC (Hyphy package).

## Supplementary information

**Additional file 1: Table 1.** Miseq run summary for the four independent Miseq runs. (N) is defined as the total number of samples included in the study. The percentage (%) of duplicated is directly related to the number of replicates out of the total number of samples in the study. The 3% of the average of reads calculated from the average number of reads per sample.

**Additional file 2: Table 2.** Two model comparisons conducted between a neutral model (M1a, M7) and a model allowing for positive selection (M2a, M8). Significance was assessed by comparing twice the difference in likelihood 2(Lb-La) between the models to a χ2-distribution (df = 2). Positively selected codons were cal-culated by Bayes Empirical Bayes (BEB) at the > 95% confidence level, by using the Effect likelihood approach (FEL) and the random effect likelihood (REL). Coincident codons are underlined. In total number of sites under se-lection (N), sites estimated at least by two approaches were counted as one. Average of ω ratio per site was estimated with SLAC.

**Additional file 3: Figure 1.** Representation of the post-glacial colonization routes followed by a blue narrow in by *R. arvalis* A) and a brown narrow in *R. temporaria* B). Black dash lines represent the potencial contact zone between the two routes approaching from the north and south. Frogs illustrations were created by A.Cortazar for this specific study.

**Additional file 4: Figure 2.** Allele frequency distribution of the Temporin, Brevinin and Pal-ustrin group of genes in 14 *R. arvalis* populations (upper row: A: Altwarm-büchen; M: Mardof; Se: Seebeckwiesen; S: Sjöhusen; T: Tvedöra; R: Räften; AÖ: Österbybruk; V: Valsbrunna; C: Crayfish/Almby; H: Holmsjön; Ny: Nydalasjön; B: Besbyn; E: Ernäs; G: Gemträsket) and 17 *R.temporaria* pop-ulations (lower row: B: Altwarmbüchen; K: Schneeren – Kuhteich; W: Oster-loh – Wienhausen; HO: Höör; SF: Sjöbo S; SL: Östra Odarslöv; Grä: Gränby; KO: Kolvia; Ö: Österbybruk; Taf: Tafteå; Gross: Grossjön; LT1: Besbyn; LT2: Mockträsket; LT3: Gemträsket; Ga: Gällivare; Le: Leipojärvi; F: Kilpis-järvi). Colour coding scheme for the alleles is given in the (Figure S2). Frogs illustrations were created by A.Cortazar for this specific study.

**Additional file 5: Figure 3.** Colour scheme for the allele frequency pie charts for nucleotide sequences represented in Additional file 4: Figure 2.

**Additional file 6: Figure 4.** Minimum Spanning Network haplotype reconstruction. Every circle represents one independent individual. In order to simplify the haplotype, network every individual in the plot is “2 stripes” separated from the other. Yellow circles represent *R. arvalis* individuals, and purple circles *R. temporaria* individuals. A: Temporin group of genes, B Brevinin group of genes.

**Additional file 7: Figure 5.** Molecular phylogram of nucleotide sequences of ranid antimicrobial peptides reconstructed with neighbor joining method for: a) Temporin, b) Brevinin and c) Palustrin. Bootstrap values from 1000 replicates greater than 50% are indicated on branches. Alleles that belong to the same group are included in the same colored square. Alleles shared by both R. arvalis and R. temporaria are shown in bold. Valid alleles were named following the nomenclature by Klein (1975) for MHC loci: a four-digit abbreviation of the species name followed by species_gene*numeration, e.g. Raar_Brev*01.

**Additional file 8: Figure 6.** Colour scheme for the amino-acid frequency pie charts represented in Additional file 9: Figure 7.

**Additional file 9: Figure 7.** Molecular phylogram based on the amino-acid sequences was reconstructed with neighbor join methods. Name of the valid allelic variant were named following the nomenclature suggested by Klein (1975) for MHC loci: a four-digit abbreviation of the species name followed by spe-cies_gene*numeration, e.g. Raar_Brev*01. Nucleotide sequences function-ally indentical were named by gene_Amino*numeration. Bootstrap values from 1000 replicates greater than 50% are indicated on branches.

**Additional file 10: Figure 8.** Minimum Spanning Network representing Temporin nucleotide variants in *R.arvalis*. Every circle represents one single individual. Populations within regions along the gradient are represented with the same colour.

**Additional file 11: Figure 9.** Minimum Spanning Network representing Temporin nucleotide variants in *R.temporaria*. Every circle represents one single indvidual. Populations within regions along the gradient are represented with the same colour.

**Additional file 12: Figure 10.** Minimum Spanning Network representing Brevinin nucleotide variants in *R.arvalis*. Every circle represents one single indvidual. Populations within regions along the gradient are represented with the same colour.

**Additional file 13: Figure 11.** Minimum Spanning Network representing Brevinin nucleotide variants in *R. temporaria*. Every circle represents one single indvidual. Populations within regions along the gradient are represented with the same colour.

**Additional file 14: Figure 12.** Minimum Spanning Network representing Palustrin nucleotide variants in *R. temporaria*. Every circle represents one single indvidual. Popu-lations within regions along the gradient are represented with the same colour.

**Additional file 15: Figure 13.** Net charge of the Acidic Propiece domain is relatively related to the net charge of the Mature Peptide domain for a) Temporin and Brevinin sequences and b) Palustrin sequences. The regression line is plotted in green and brown, respectively in A) and B).

**Additional file 16: Figure 14.** Antimicrobial peptide alignment. The Signal Peptide is rep-resented in blue, the Acidic Propiece in black and the Mature Peptide in red. Codon under selection were marked with an asterisk (*). Codons under selection detected at least by two different methods are colored in yellow. A) represent Temporin-Brevinin group of genes and B) palustrin group of genes, respectively.

## Data Availability

The datasets used and/or analysed during the current study are available from the corresponding author on request.

## Abbreviations

AMPs: Antimicrobial peptides
DOC: Degree of change
MHC: Major histocompatibility complex

## References

[1] Wood PJ. Understanding immunology. Pearson Education. 2006.

[2] Janeway CA, Travers P, Walport M, Shlomchik M (2001). Immunobiology: the immune system in health and disease, vol. 2.

[3] Corsaro C, Scalia M, Leotta N, Mondio F, Sichel G (2000). Characterisation of Kupffer cells in some Amphibia. J Anat.

[4] Horton J, Horton T, Dzialo R, Gravawr I, Minter R, Ritchie P, Ganluna L, Watson M, Caopet M (1998). T-cell and natural killer cell development in thymectomized Xenopus. Immunol Rev.

[5] Apponyi MA, Pukala TL, Brinkworth CS, Maselli VM, Bowie JH, Tyler MJ, Booker GW, Wallace JC, Carver JA, Separovic F (2004). Host-defence peptides of Australian anurans: structure, mechanism of action and evolutionary significance. Peptides.

[6] Duda TF, Vanhoye D, Nicolas P (2002). Roles of diversifying selection and coordinated evolution in the evolution of amphibian antimicrobial peptides. Mol Biol Evol.

[7] Tennessen JA (2005). Molecular evolution of animal antimicrobial peptides: widespread moderate positive selection. J Evol Biol.

[8] Brown KL, Hancock REW (2006). Cationic host defense (antimicrobial) peptides. Curr Opin Immunol.

[9] Groenink J, Walgreen-Weterings E, van't Hof W, Veerman ECI, Amerongen AVN (1999). Cationic amphipathic peptides, derived from bovine and human lactoferrins, with antimicrobial activity against oral pathogens. FEMS Microbiol Lett.

[10] Huang YB, Huang JF, Chen YX (2010). Alpha-helical cationic antimicrobial peptides: relationships of structure and function. Protein Cell.

[11] Gosset CC, Do Nascimento J, Auge M-T, Bierne N (2014). Evidence for adaptation from standing genetic variation on an antimicrobial peptide gene in the mussel *Mytilus edulis*. Mol Ecol.

[12] Halldorsdottir K, Arnason E. Trans-species polymorphism at antimicrobial innate immunity cathelicidin genes of Atlantic cod and related species. Peerj. 2015;3. 10.7717/peerj.976. 10.7717/peerj.976 PMC4451034 26038731

[13] Robertson LS, Cornman RS (2014). Transcriptome resources for the frogs *Lithobates clamitans* and *Pseudacris regilla*, emphasizing antimicrobial peptides and conserved loci for phylogenetics. Mol Ecol Resour.

[14] Muncaster S, Kraakman K, Gibbons O, Mensink K, Forlenza M, Jacobson G, Bird S: doi:10.1016/j.dci.2017.04.014. 10.1016/j.dci.2017.04.014 28433529

[15] Cagliani R, Fumagalli M, Riva S, Pozzoli U, Comi GP, Menozzi G, Bresolin N, Sironi M (2008). The signature of long-standing balancing selection at the human defensin beta-1 promoter. Genome Biol.

[16] Vanhoye D, Bruston F, Nicolas P, Amiche M (2003). Antimicrobial peptides from hylid and ranin frogs originated from a 150-million-year-old ancestral precursor with a conserved signal peptide but a hypermutable antimicrobial domain. Eur J Biochem.

[17] Tennessen JA, Blouin MS (2008). Balancing selection at a frog antimicrobial peptide locus: fluctuating immune effector alleles?. Mol Biol Evol.

[18] Bahar A, Ren D (2013). Antimicrobial peptides. Pharmaceuticals.

[19] Woodhams DC, Voyles J, Lips KR, Carey C, Rollins-Smith LA (2006). Predicted disease susceptibility in a panamanian amphibian assemblage based on skin peptide defenses. J Wildl Dis.

[20] Woodhams DC, Rollins-Smith LA, Carey C, Reinert L, Tyler MJ, Alford RA (2006). Population trends associated with skin peptide defenses against chytridiomycosis in Australian frogs. Oecologia.

[21] Scheele BC, Pasmans F, Skerratt LF, Berger L, Martel A, Beukema W, Acevedo AA, Burrowes PA, Carvalho T, Catenazzi A (2019). Amphibian fungal panzootic causes catastrophic and ongoing loss of biodiversity. Science.

[22] Chapman JR, Hill T, Unckless RL (2019). Balancing selection drives the maintenance of genetic variation in Drosophila antimicrobial peptides. Genome Biol Evol.

[23] Conlon JM, Kolodziejek J, Nowotny N (2004). Antimicrobial peptides from ranid frogs: taxonomic and phylogenetic markers and a potential source of new therapeutic agents. BBA-Proteins Proteomics.

[24] Marcocci ME, Amatore D, Villa S, Casciaro B, Aimola P, Franci G, Grieco P, Galdiero M, Palamara AT, Mangoni ML (2018). The amphibian antimicrobial peptide Temporin B inhibits in vitro herpes simplex virus 1 infection. Antimicrob Agents Chemother.

[25] Selsted ME, Ouellette AJ (2005). Mammalian defensins in the antimicrobial immune response. Nat Immunol.

[26] Hellgren O, Sheldon BC (2011). Locus-specific protocol for nine different innate immune genes (antimicrobial peptides: beta-defensins) across passerine bird species reveals within-species coding variation and a case of trans-species polymorphisms. Mol Ecol Resour.

[27] Woodhams DC, Kenyon N, Bell SC, Alford RA, Chen S, Billheimer D, Shyr Y, Rollins-Smith LA (2010). Adaptations of skin peptide defences and possible response to the amphibian chytrid fungus in populations of Australian green-eyed treefrogs, Litoria genimaculata. Divers Distributions.

[28] Mina AE, Ponti AK, Woodcraft NL, Johnson EE, Saporito RA (2015). Variation in alkaloid-based microbial defenses of the dendrobatid poison frog *Oophaga pumilio*. Chemoecology.

[29] Davis LR, Klonoski K, Rutschow HL, Van Wijk KJ, Sun Q, Haribal MM, Saporito RA, Vega A, Rosenblum EB, Zamudio KR (2016). Host defense skin peptides vary with color pattern in the highly polymorphic red-eyed Treefrog. Front Ecol Evol.

[30] Lazzaro BP (2008). Natural selection on the Drosophila antimicrobial immune system. Curr Opin Microbiol.

[31] Tennessen JA, Blouin MS (2007). Selection for antimicrobial peptide diversity in frogs leads to gene duplication and low allelic variation. J MoEvol.

[32] Bijlsma R, Loeschcke V (2012). Genetic erosion impedes adaptive responses to stressful environments. Evol Appl.

[33] Hewitt GM (1999). Post-glacial re-colonization of European biota. Biol J Linn Soc.

[34] Hampe A, Petit RJ (2005). Conserving biodiversity under climate change: the rear edge matters. Ecol Lett.

[35] Strand AE, Williams LM, Oleksiak MF, Sotka EE (2012). Can diversifying selection be distinguished from history in geographic clines? A population genomic study of killifish (*Fundulus heteroclitus*). PLoS One.

[36] de Lafontaine G, Napier JD, Petit RJ, Hu FS (2018). Invoking adaptation to decipher the genetic legacy of past climate change. Ecology.

[37] Palo JU, Schmeller DS, Laurila A, Primmer CR, Kuzmin SL, Merila J (2004). High degree of population subdivision in a widespread amphibian. Mol Ecol.

[38] Jansen van Rensburg A. The genomics of adaptation to climate across latitude and elevation in the European common frog: University of Zurich; 2018. https://www.zora.uzh.ch/id/eprint/153943/1/153943.pdf.

[39] Knopp T, Merila J (2009). The postglacial recolonization of northern Europe by *Rana arvalis* as revealed by microsatellite and mitochondrial DNA analyses. Heredity.

[40] Cortázar-Chinarro M, Lattenkamp EZ, Meyer-Lucht Y, Luquet E, Laurila A, Höglund J (2017). Drift, selection, or migration? Processes affecting genetic differentiation and variation along a latitudinal gradient in an amphibian. BMC Evol Biol.

[41] Kirkpatrick M, Barton NH (1997). Evolution of a species' range. Am Nat.

[42] Johansson M, Primmer CR, Merila J (2006). History vs. current demography: explaining the genetic population structure of the common frog (*Rana temporaria*). Mol Ecol.

[43] Rödin‐Mörch P, Luquet E, Meyer‐Lucht Y, Richter‐Boix A, Höglund J, Laurila A. Latitudinal divergence in a widespread amphibian: Contrasting patterns of neutral and adaptive genomic variation. Mol Ecol. 2019;28(12):2996-3011. 10.1111/mec.15132 31134695

[44] Eckert CG, Samis KE, Lougheed SC (2008). Genetic variation across species' geographical ranges: the central-marginal hypothesis and beyond. Mol Ecol.

[45] Lighten J, Van Oosterhout C, Paterson IG, McMullan M, Bentzen P (2014). Ultra-deep Illumina sequencing accurately identifies MHC class IIb alleles and provides evidence for copy number variation in the guppy (*Poecilia reticulata*). Mol Ecol Res.

[46] Yang Z (2007). PAML 4: phylogenetic analysis by maximum likelihood. Mol Biol Evol.

[47] Cortazar-Chinarro M, Meyer-Lucht Y, Laurila A, Höglund J (2018). Signatures of historical selection on MHC reveal different selection patterns in the moor frog (*Rana arvalis*). Immunogenetics.

[48] van Rensburg AJ, Cortazar-Chinarro M, Laurila A, Van Buskirk J. Adaptive genomic variation associated with environmental gradients along a latitudinal cline in Rana temporaria. 2018. bioRxiv:427872.

[49] Lucht Y, Luquet E, Jóhannesdóttir F, Rödin‐Mörch P, Quintela M, Richter‐Boix A, ...Laurila A. Genetic basis of amphibian larval development along a latitudinal gradient: Gene diversity, selection and links with phenotypic variation in transcription factor C/EBP‐1. Mol Ecol. 2019;28(11):2786–801. 10.1111/mec.15123 31067349

[50] Schemske DW, Mittelbach GG, Cornell HV, Sobel JM, Roy K (2009). Is there a latitudinal gradient in the importance of biotic interactions?. Annu Rev Ecol Evol Syst.

[51] Anstett DN, Ahern JR, Johnson MT, Salminen JP (2018). Testing for latitudinal gradients in defense at the macroevolutionary scale. Evolution.

[52] Guernier V, Hochberg ME, Guegan JF (2004). Ecology drives the worldwide distribution of human diseases. PLoS Biol.

[53] Morand S, Walther BA (2018). Individualistic values are related to an increase in the outbreaks of infectious diseases and zoonotic diseases. Sci Rep.

[54] Somero GN (2010). The physiology of climate change: how potentials for acclimatization and genetic adaptation will determine 'winners' and 'losers'. J Exp Biol.

[55] Hoglund J, Wengstrom A, Rogell B, Meyer-Lucht Y (2015). Low MHC variation in isolated island populations of the Natterjack toad (*Bufo calamita*). Con Gen.

[56] Skerratt LF, Berger L, Speare R, Cashins S, McDonald KR, Phillott AD, Hines HB, Kenyon N (2007). Spread of chytridiomycosis has caused the rapid global decline and extinction of frogs. Ecohealth.

[57] Rödder D, Kielgast J, Bielby J, Schmidtlein S, Bosch J, Garner TW, Veith M, Walker S, Fisher M, Lötters S (2009). Global amphibian extinction risk assessment for the panzootic chytrid fungus. Diversity.

[58] O’hanlon SJ, Rieux A, Farrer RA, Rosa GM, Waldman B, Bataille A, Kosch TA, Murray KA, Brankovics B, Fumagalli M (2018). Recent Asian origin of chytrid fungi causing global amphibian declines. Science.

[59] Karvemo S, Meurling S, Berger D, Hoglund J, Laurila A (2018). Effects of host species and environmental factors on the prevalence of *Batrachochytrium dendrobatidis* in northern Europe. PLoS One.

[60] Meurling S. The response in native wildlife to an invading pathogen: Swedish amphibians and Batracho-chytrium dendrobatidis. PhD theis: Uppsala University; 2019. http://www.diva-portal.org/smash/record.jsf?pid=diva2%3A1369696&dswid=-1094.

[61] Patrelle C, Miaud C, Cristina N, Kullberg P, Merilä J (2012). Chytrid fungus screening in a population of common frogs from northern Finland. Herpetol Rev.

[62] Esposito E (2018). Analysis of antimicrobial peptide efficacy against chytridiomycosis from skin secretions of Columbia spotted frogs (*Lithobates luteiventris*). PhD Thesis.

[63] Flechas SV, Acosta-González A, Escobar LA, Kueneman JG, Sánchez-Quitian ZA, Parra-Giraldo CM, Rollins-Smith LA, Reinert LK, Vredenburg VT, Amézquita A (2019). Microbiota and skin defense peptides may facilitate coexistence of two sympatric Andean frog species with a lethal pathogen. ISME J.

[64] Rollins-Smith LA, Doersam JK, Longcore JE, Taylor SK, Shamblin JC, Carey C, Zasloff MA (2002). Antimicrobial peptide defenses against pathogens associated with global amphibian declines. Dev Comp Immunol.

[65] Rollins-Smith LA, Conlon JM (2005). Antimicrobial peptide defenses against chytridiomycosis, an emerging infectious disease of amphibian populations. Dev Comp Immunol.

[66] Maxwell A, Morrison G, Dorin J (2003). Rapid sequence divergence in mammalian β-defensins by adaptive evolution. Mol Immunol.

[67] Zhang J (2003). Evolution by gene duplication: an update. Trends Ecol Evol.

[68] Burri R, Salamin N, Studer RA, Roulin A, Fumagalli L (2010). Adaptive divergence of ancient gene duplicates in the avian MHC class II beta. Mol Biol Evol.

[69] Bracamonte SE, Baltazar-Soares M, Eizaguirre C (2015). Characterization of MHC class II genes in the critically endangered European eel (*Anguilla anguilla*). Conserv Genet Resour.

[70] Talarico L, Babik W, Marta S, Mattoccia M. Genetic drift shaped MHC IIB diversity of an endangered anuran species within the Italian glacial refugium. J Zool. 2019;307(1):61-70.

[71] Fan WM, Kasahara M, Gutknecht J, Klein D, Mayer WE, Jonker M, Klein J (1989). Shared class-II MHC polymorphisms between humans and chimpanzees. Hum Immunol.

[72] Strand T, Westerdahl H, Hoeglund J, Alatalo RV, Siitari H (2007). The Mhc class II of the black grouse (Tetrao tetrix) consists of low numbers of B and Y genes with variable diversity and expression. Immunogenetics.

[73] Eimes JA, Townsend AK, Sepil I, Nishiumi I, Satta Y. Patterns of evolution of MHC class II genes of crows (Corvus) suggest trans-species polymorphism. PeerJ. 2015;3:e853. 10.7717/peerj.853. 10.7717/peerj.853 PMC4369332 25802816

[74] Veith M, Kosuch J, Vences M (2003). Climatic oscillations triggered post-Messinian speciation of Western Palearctic brown frogs (Amphibia, Ranidae). Mol Phylogenet Evol.

[75] Yuan Z-Y, Zhou W-W, Chen X, Poyarkov NA, Chen H-M, Jang-Liaw N-H, Chou W-H, Matzke NJ, Iizuka K, Min M-S (2016). Spatiotemporal diversification of the true frogs (genus Rana): a historical framework for a widely studied group of model organisms. Syst Biol.

[76] Hughes AL, Yeager M (1997). Coordinated amino acid changes in the evolution of mammalian defensins. J Mol Evol.

[77] Sommer S (2005). The importance of immune gene variability (MHC) in evolutionary ecology and conservation. Front Zool.

[78] Piertney SB, Oliver MK (2006). The evolutionary ecology of the major histocompatibility complex. Heredity.

[79] Spurgin LG, Richardson DS (2010). How pathogens drive genetic diversity: MHC, mechanisms and misunderstandings. Proc R Soc Lond B Biol Sci.

[80] Sanchez-Mazas A, Cerny V, Di D, Buhler S, Podgorna E, Chevallier E, Brunet L, Weber S, Kervaire B, Testi M (2017). The HLA-B landscape of Africa: signatures of pathogen-driven selection and molecular identification of candidate alleles to malaria protection. Mol Ecol.

[81] Fog K, Schmedes A, Rosenørn de Lasson D (1997). Nordens padder og krybdyr.

[82] Gosner KL (1960). A simplified table for staging anuran embryos and larvae with notes on identification. Herpetologica.

[83] Simmaco M, Mignogna G, Canofeni S, Miele R, Mangoni ML, Barra D (1996). Temporins, antimicrobial peptides from the European red frog Rana temporaria. Eur J Biochem.

[84] Koyama T, Conlon JM, Iwamuro S (2011). Molecular Cloning and Characterization of cDNAs Encoding Biosynthetic Precursors for the Antimicrobial Peptides Japonicin-1Ja, Japonicin-2Ja, and Temporin-1Ja in the Japanese Brown Frog, *Rana japonica*. Zool Sci.

[85] Chen TB, Zhou M, Rao PF, Walker B, Shaw C (2006). The Chinese bamboo leaf odorous frog (*Rana Odorrana versabilis*) and north American Rana frogs share the same families of skin antimicrobial peptides. Peptides.

[86] Zhou M, Wang L, Owens DE, Chen TB, Walker B, Shaw C (2007). Rapid identification of precursor cDNAs encoding five structural classes of antimicrobial peptides from pickerel frog (*Rana palustris*) skin secretion by single step "shotgun" cloning. Peptides.

[87] Magoč T, Salzberg SL (2011). FLASH: fast length adjustment of short reads to improve genome assemblies. Bioinformatics.

[88] Stuglik MT, Radwan J, Babik W (2011). jMHC: software assistant for multilocus genotyping of gene families using next-generation amplicon sequencing. Mol Ecol Res.

[89] Babik W, Pabijan M, Arntzen JW, Cogalniceanu D, Durka W, Radwan J (2009). Long-term survival of a urodele amphibian despite depleted major histocompatibility complex variation. Mol Ecol.

[90] Galan M, Guivier E, Caraux G, et al. A 454 multiplex sequencing method for rapid and reliable genotyping of highly polymorphic genes in large-scale studies. BMC Genomics. 2010;11:296. 10.1186/1471-2164-11-296. 10.1186/1471-2164-11-296 PMC2876125 20459828

[91] Lighten J, Van Oosterhout C, Bentzen P (2014). Critical review of NGS analyses for de novo genotyping multigene families. Mol Ecol.

[92] Kumar S, Stecher G, Tamura K (2016). MEGA7: molecular evolutionary genetics analysis version 7.0 for bigger datasets. Mol Biol Evol.

[93] Klein J. Many Questions (and Almost No Answers) about the Phylogenetic Origin of the Major Histocompatibility Complex. In: Hildemann WH, Benedict AA. (eds) Immunologic Phylogeny. Advances in Experimental Medicine and Biology, vol 64. Boston: Springer; 1975. 10.1007/978-1-4684-3261-9_47 1199884

[94] Excoffier L, Lischer HEL (2010). Arlequin suite ver 3.5: a new series of programs to perform population genetics analyses under Linux and windows. Mol Ecol Resour.

[95] Bates D, Sarkar D, Bates MD, Matrix L (2007). The lme4 package. R package version.

[96] Leigh JW, Bryant D (2015). popart: full-feature software for haplotype network construction. Methods Ecol Evol.

[97] Ginestet C (2011). ggplot2: elegant graphics for data analysis. J R Stat Soc Ser A-Stat Soc.

[98] Librado P, Rozas J (2009). DnaSP v5: a software for comprehensive analysis of DNA polymorphism data. Bioinformatics.

[99] Pond SLK, Frost SDW, Muse SV (2005). HyPhy: hypothesis testing using phylogenies. Bioinformatics.

[100] Weaver S, Shank SD, Spielman SJ, Li M, Muse SV, Kosakovsky Pond SL (2018). Datamonkey 2.0: a modern web application for characterizing selective and other evolutionary processes. Mol Biol Evol.

