# Supplementary material for: Antimicrobial peptide and sequence variation along a latitudinal gradient in two anurans
Source: BMC Genet. 2020 Mar 30;21:38. doi: 10.1186/s12863-020-00839-1 (PMC7106915; doi:10.1186/s12863-020-00839-1)
Supplement: Supplementary file 1 — Additional file 1: Table 1. Miseq run summary for the four independent Miseq runs. (N) is defined as the total number of samples included in the study. The percentage (%) of duplicated is directly related to the number of replicates out of the total number of samples in the study. The 3% of the average of reads calculated from the average number of reads per sample. [file 12863_2020_839_MOESM1_ESM.pdf]

**Samples**	
**(N) *R. arvalis***	150
**(N) *R. temporaria***	170
Miseq Run 1	
**Temporin**	
**Rana arvalis**	
Total number of reads	3.704.418
Total number of replicates	40
Average number of reads per sample	20580
3% of the avergae of reads	617
% percentage of duplicates	22
Miseq Run2	
**Temporin**	
**Rana temporaria**	
Total number of reads	4.769.171
number of replicates	54
Average number of reads per sample	18703
3% of the avergae of reads	561
% percentage of duplicates	21
Miseq Run 3	
**Brevinin**	
Rana temporia replicates	21
Rana arvalis replicates	42
Total number of reads	2.597.554
Average number of reads per sample	6543
3% of the avergae of reads	196
% percentage of duplicates	16
Miseq Run 4	
**Palustrin**	
Rana temporia replicates	43
Rana arvalis replicates	25
Total number of reads	5.124.580
Average number of reads per sample	15343
3% of the avergae of reads	460
% percentage of duplicates	20
