# Supplementary material for: Antimicrobial peptide and sequence variation along a latitudinal gradient in two anurans
Source: BMC Genet. 2020 Mar 30;21:38. doi: 10.1186/s12863-020-00839-1 (PMC7106915; doi:10.1186/s12863-020-00839-1)
Supplement: Supplementary file 2 — Additional file 2: Table 2. Two model comparisons conducted between a neutral model (M1a, M7) and a model allowing for positive selection (M2a, M8). Significance was assessed by comparing twice the difference in likelihood 2(Lb-La) between the models to a χ2-distribution (df = 2). Positively selected codons were cal-culated by Bayes Empirical Bayes (BEB) at the > 95% confidence level, by using the Effect likelihood approach (FEL) and the random effect likelihood (REL). Coincident codons are underlined. In total number of sites under se-lection (N), sites estimated at least by two approaches were counted as one. Average of ω ratio per site was estimated with SLAC. [file 12863_2020_839_MOESM2_ESM.pdf]

		PALM (codeML)			FEL	REL			
**Locus**	**Model Comparison**	**2($L_b - L_a$)**	**p**	**dN/dS >1**	**dN/dS >1**	**dN/dS >1**	**N**	**Sites located in the Mature Peptide**	**ω**
Temporin-Brevinin	M1a-M2a	28.577578	<0.001	21,44,47,49, 50,51,56	3,40,43	47,49,50	14	12	0.651
	M7-M8	35.54987	<0.001	3,21,40,41,43,44,45, 46,47,48,49,50,51,56					
Palustrin	M1a-M2a	4E-06	<0.05	none	none	none	4	4	0.551
	M7-M8	0.084392	<0.05	23,26,29,33					
