# Supplementary figures and images for: Antimicrobial peptide and sequence variation along a latitudinal gradient in two anurans
Source: BMC Genet. 2020 Mar 30;21:38. doi: 10.1186/s12863-020-00839-1 (PMC7106915; doi:10.1186/s12863-020-00839-1)

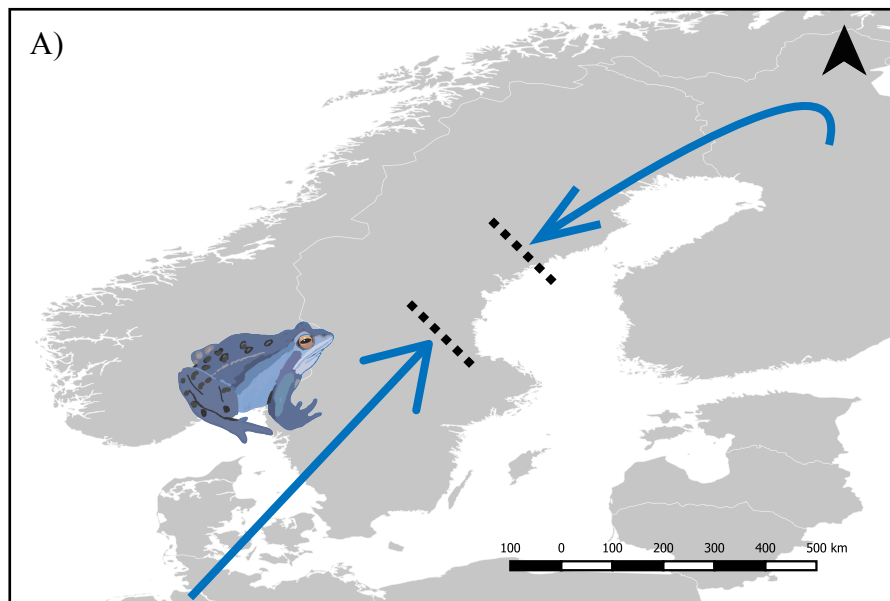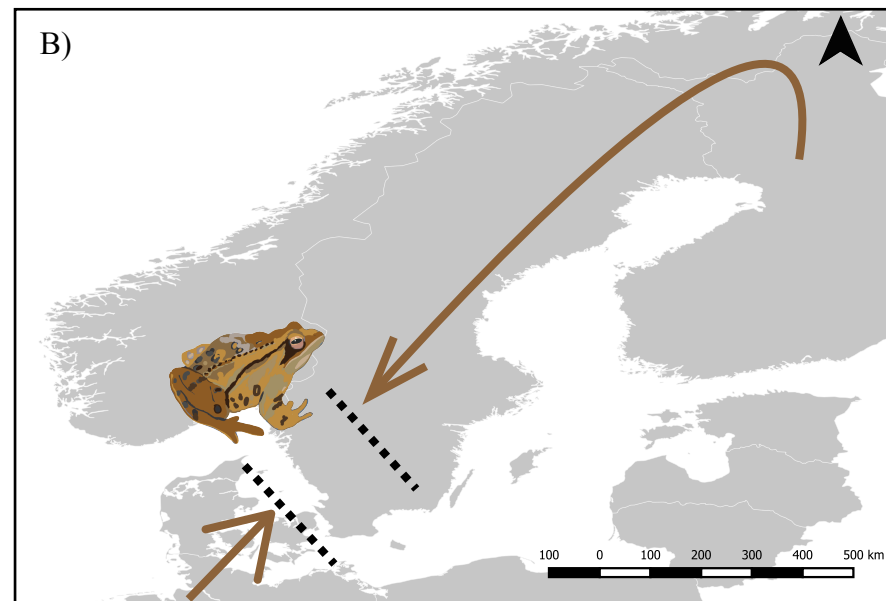

Supplement: Supplementary file 3 — Additional file 3: Figure 1. Representation of the post-glacial colonization routes followed by a blue narrow in by R. arvalis A) and a brown narrow in R. temporaria B). Black dash lines represent the potencial contact zone between the two routes approaching from the north and south. Frogs illustrations were created by A.Cortazar for this specific study. [file 12863_2020_839_MOESM3_ESM.pdf]

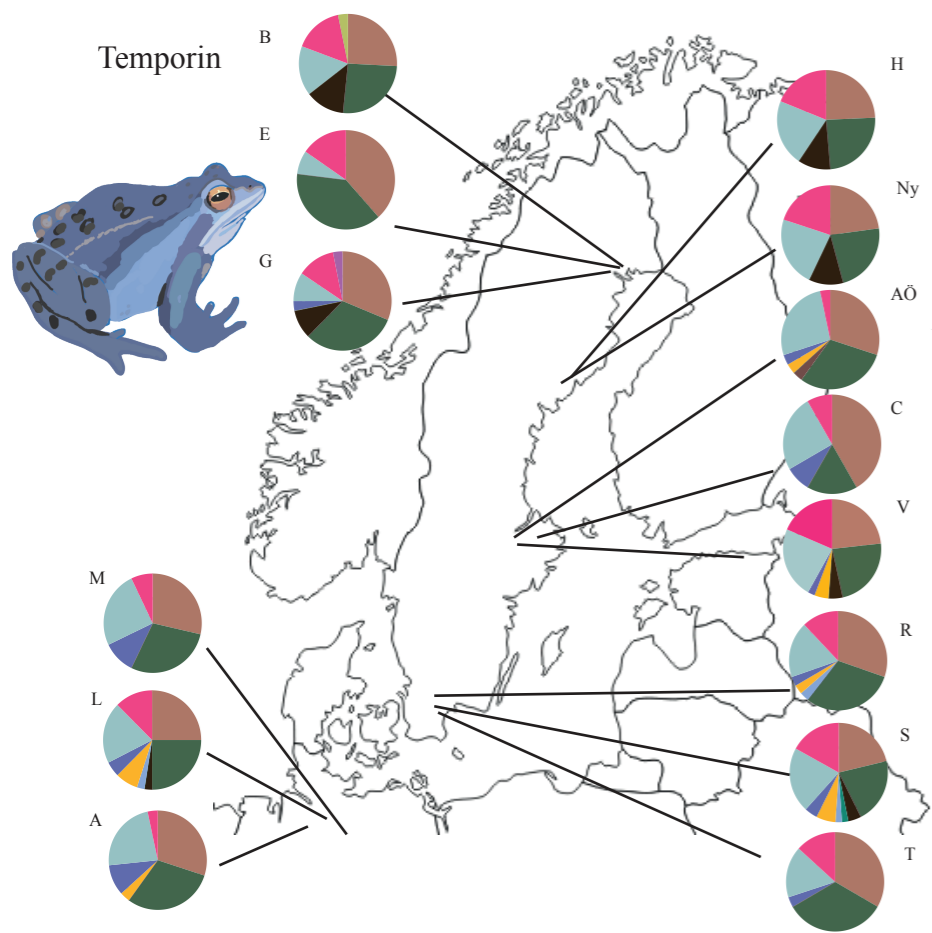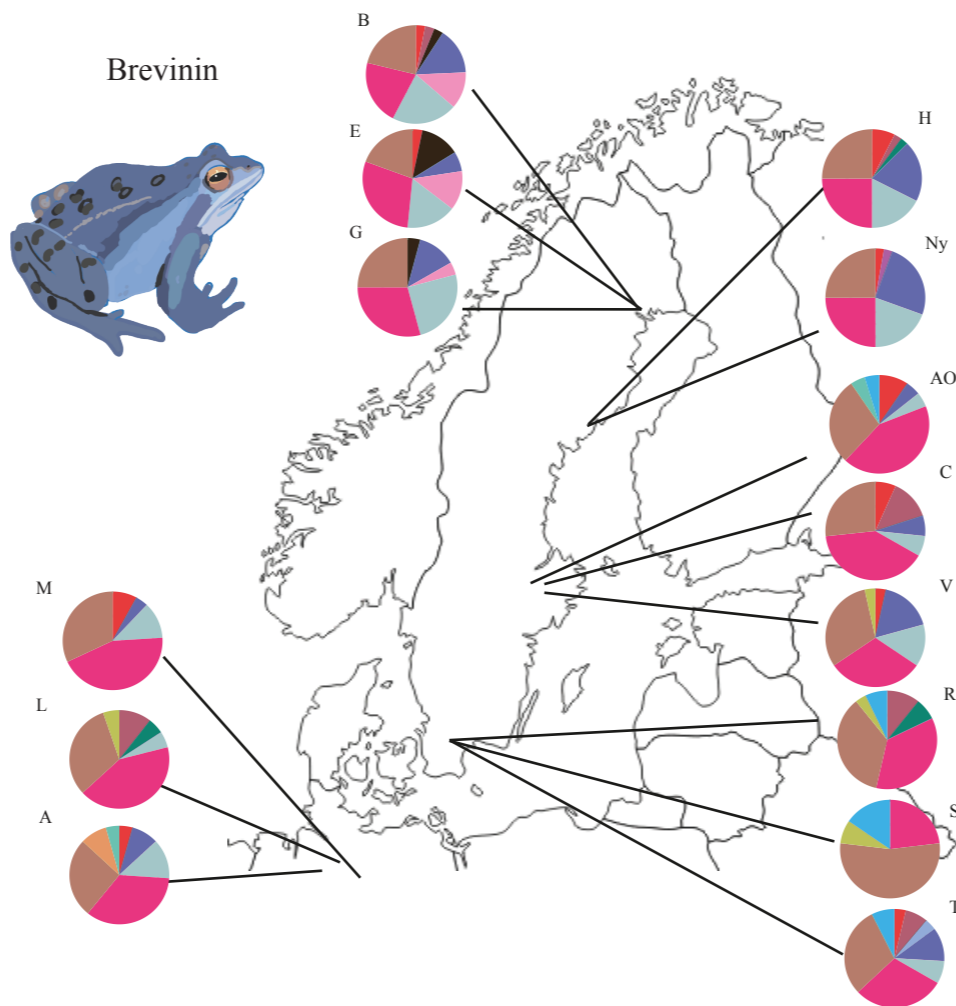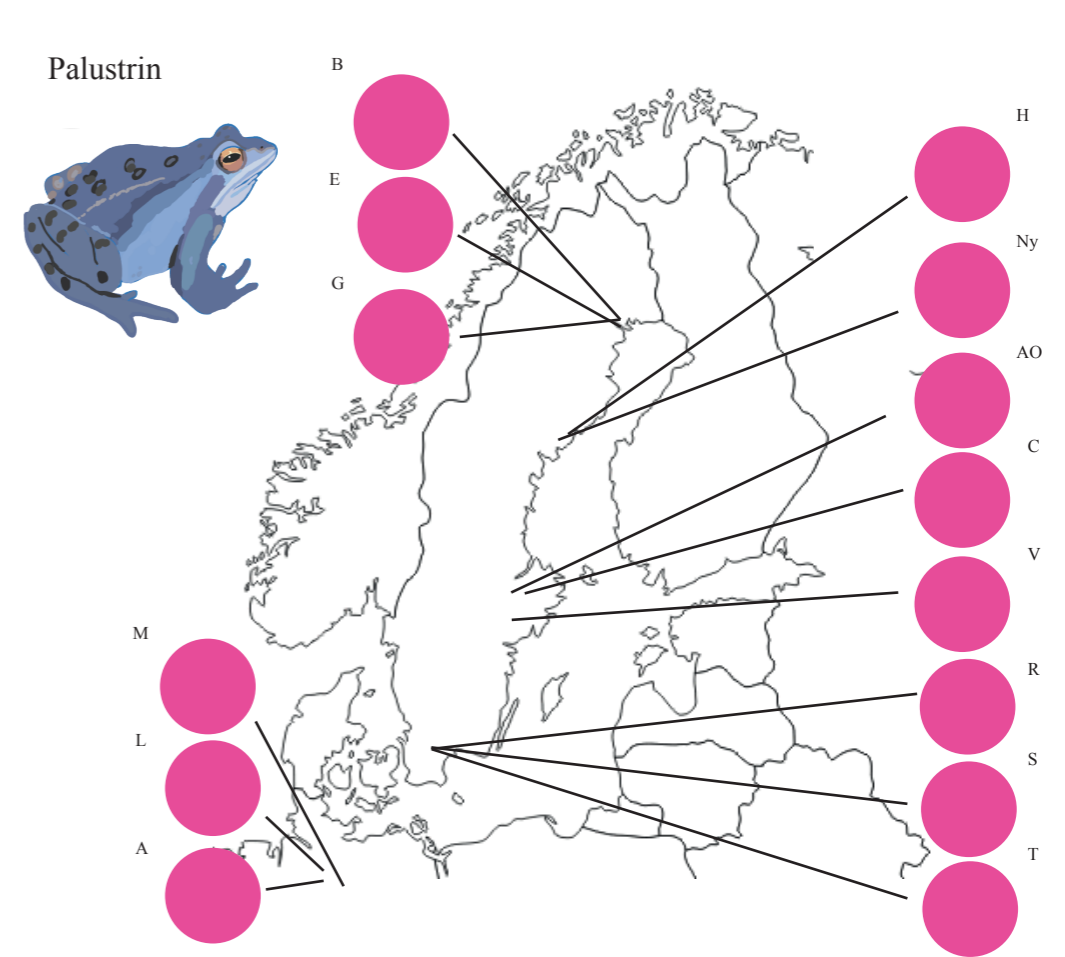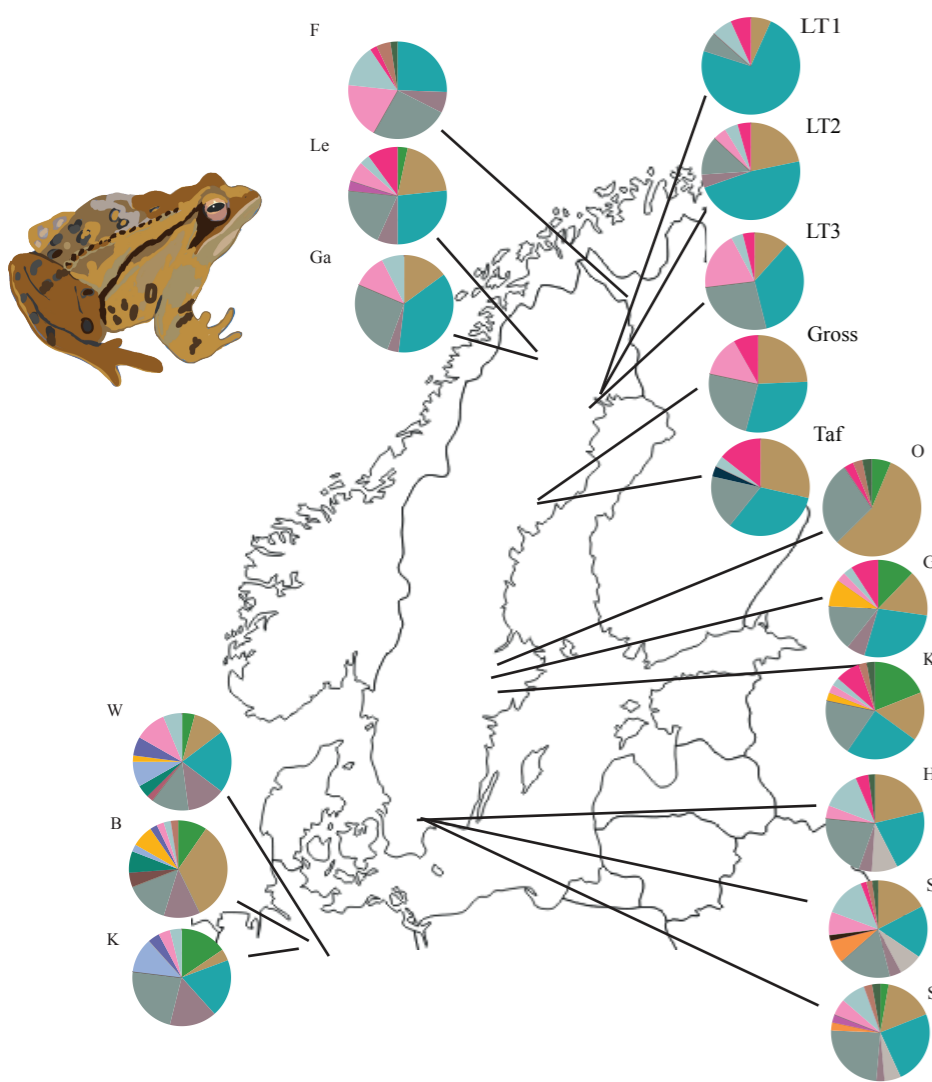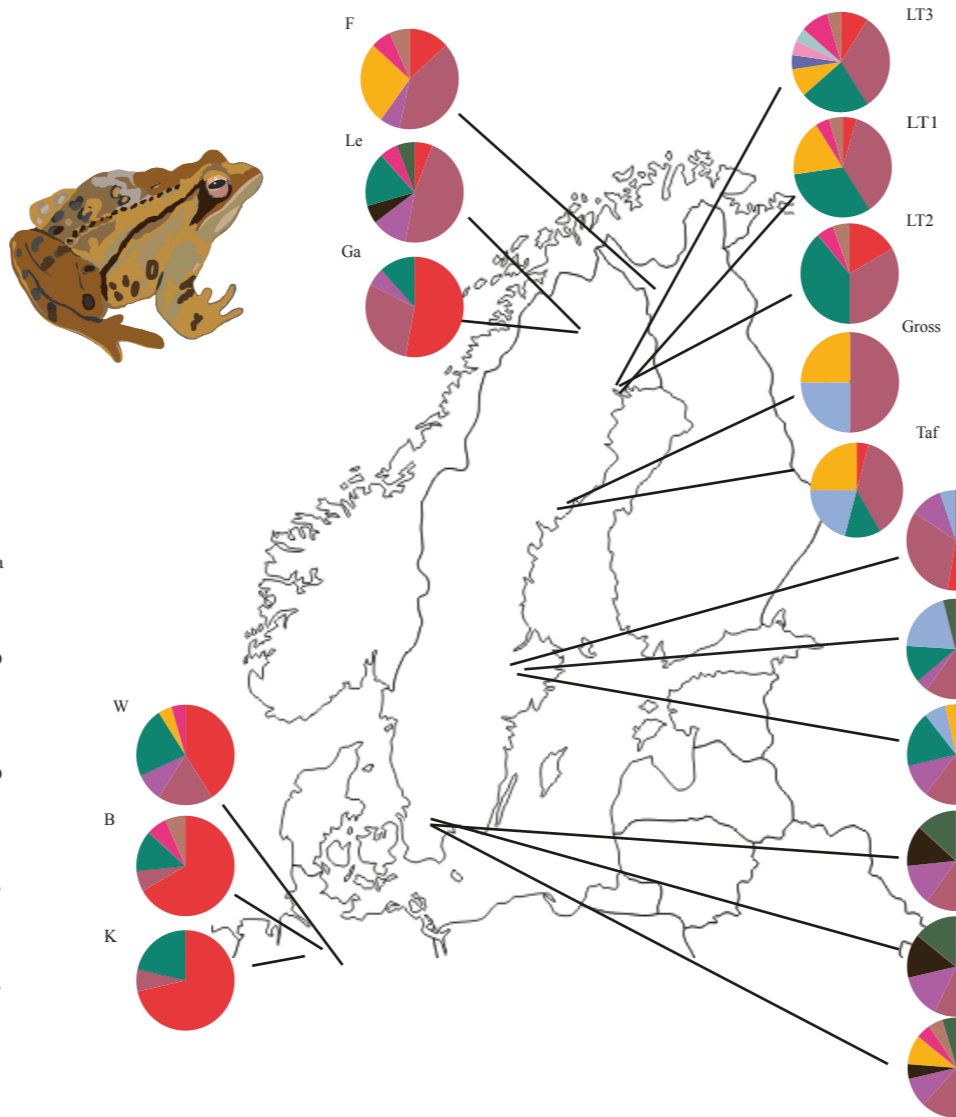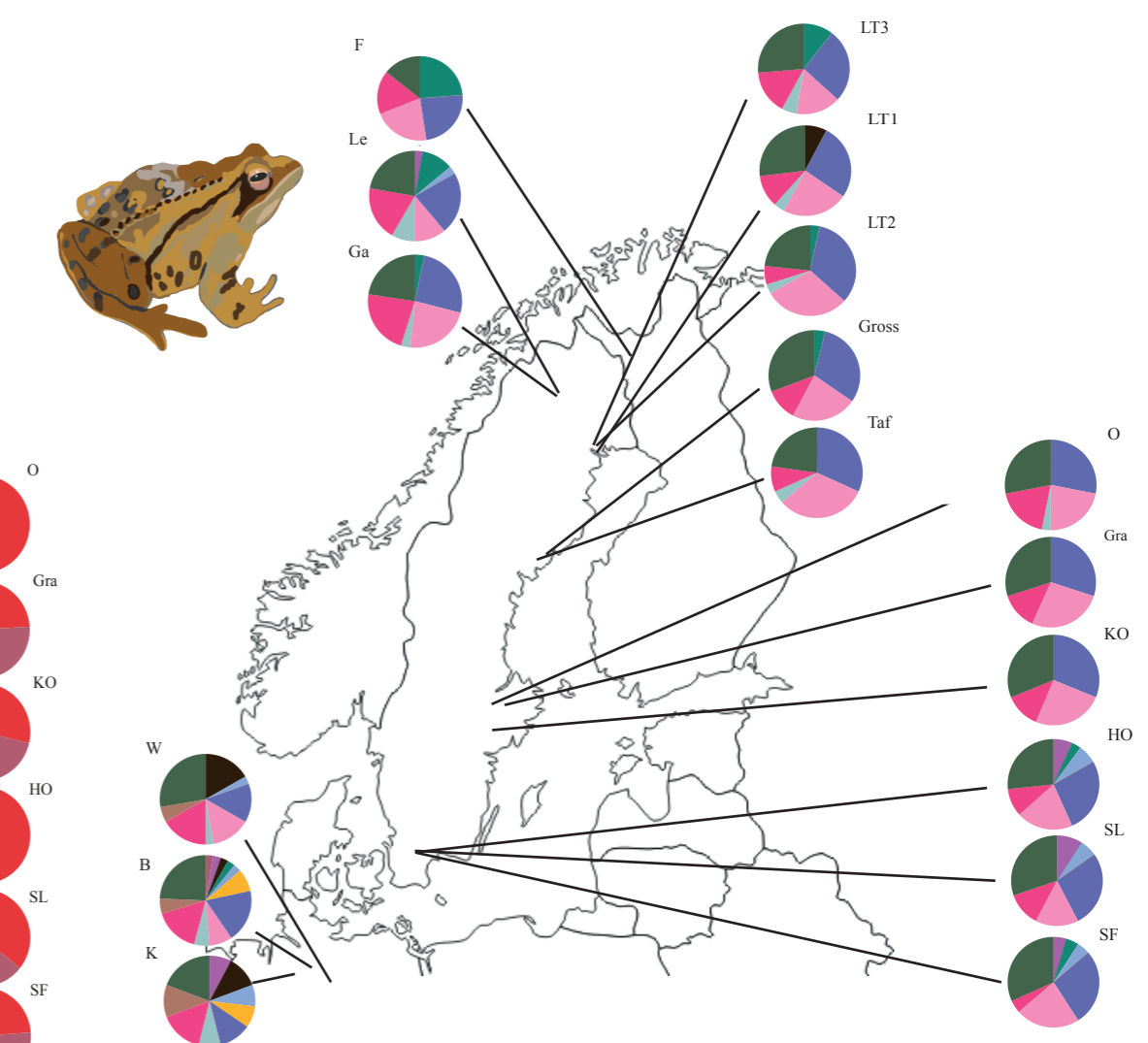

Supplement: Supplementary file 4 — Additional file 4: Figure 2. Allele frequency distribution of the Temporin, Brevinin and Pal-ustrin group of genes in 14 R. arvalis populations (upper row: A: Altwarm-büchen; M: Mardof; Se: Seebeckwiesen; S: Sjöhusen; T: Tvedöra; R: Räften; AÖ: Österbybruk; V: Valsbrunna; C: Crayfish/Almby; H: Holmsjön; Ny: Nydalasjön; B: Besbyn; E: Ernäs; G: Gemträsket) and 17 R.temporaria pop-ulations (lower row: B: Altwarmbüchen; K: Schneeren – Kuhteich; W: Oster-loh – Wienhausen; HO: Höör; SF: Sjöbo S; SL: Östra Odarslöv; Grä: Gränby; KO: Kolvia; Ö: Österbybruk; Taf: Tafteå; Gross: Grossjön; LT1: Besbyn; LT2: Mockträsket; LT3: Gemträsket; Ga: Gällivare; Le: Leipojärvi; F: Kilpis-järvi). Colour coding scheme for the alleles is given in the (Figure S2). Frogs illustrations were created by A.Cortazar for this specific study. [file 12863_2020_839_MOESM4_ESM.pdf]

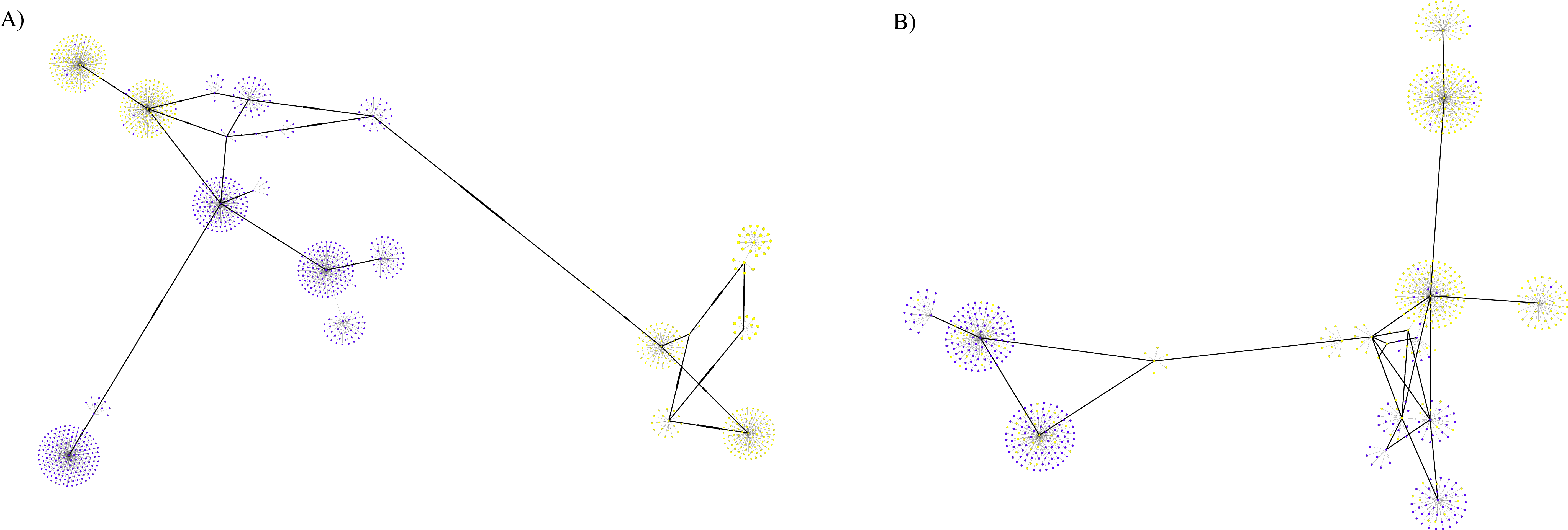

Supplement: Supplementary file 6 — Additional file 6: Figure 4. Minimum Spanning Network haplotype reconstruction. Every circle represents one independent individual. In order to simplify the haplotype, network every individual in the plot is “2 stripes” separated from the other. Yellow circles represent R. arvalis individuals, and purple circles R. temporaria individuals. A: Temporin group of genes, B Brevinin group of genes. [file 12863_2020_839_MOESM6_ESM.png]

A) Temporin

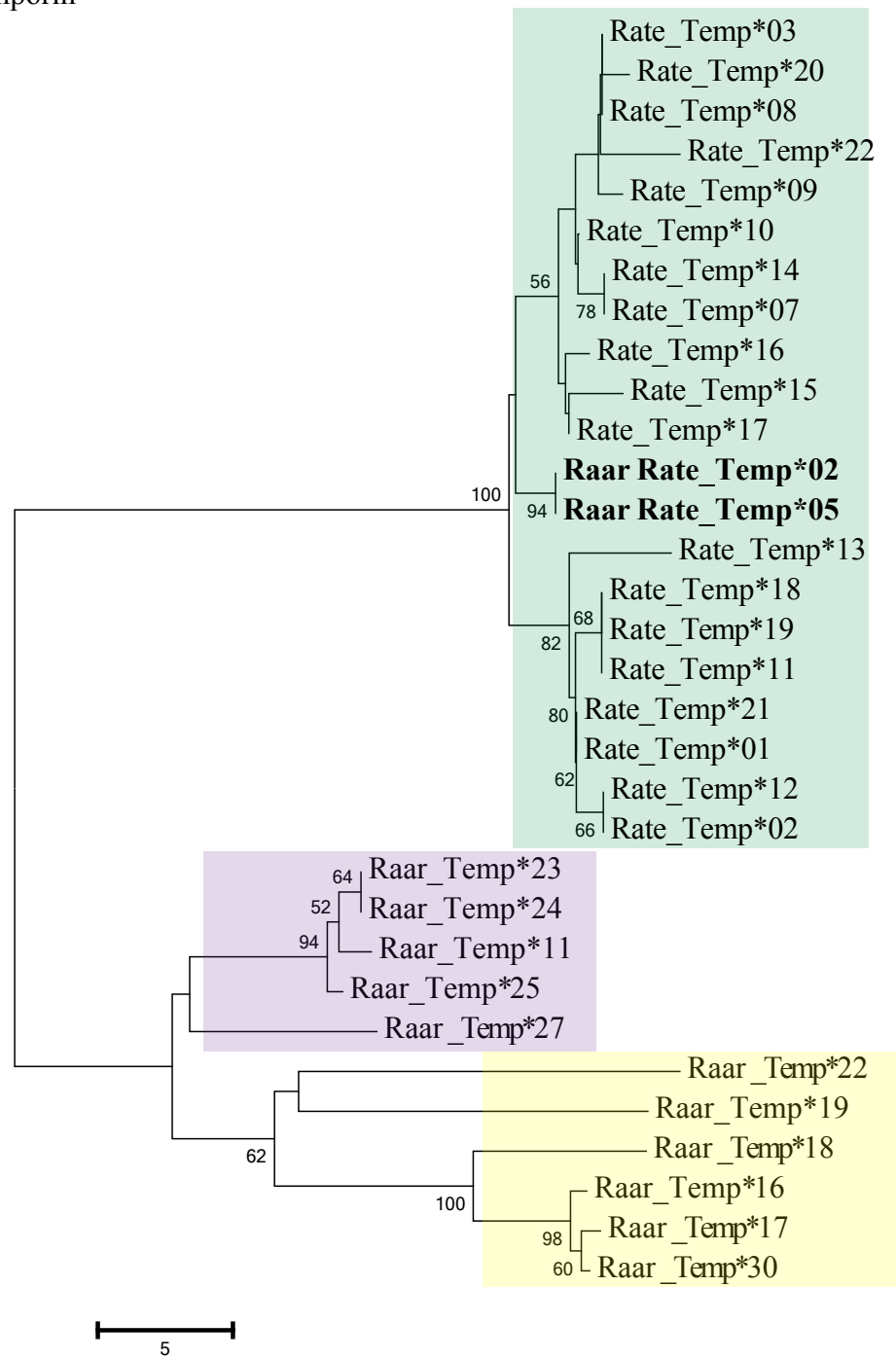

B) Brevinin

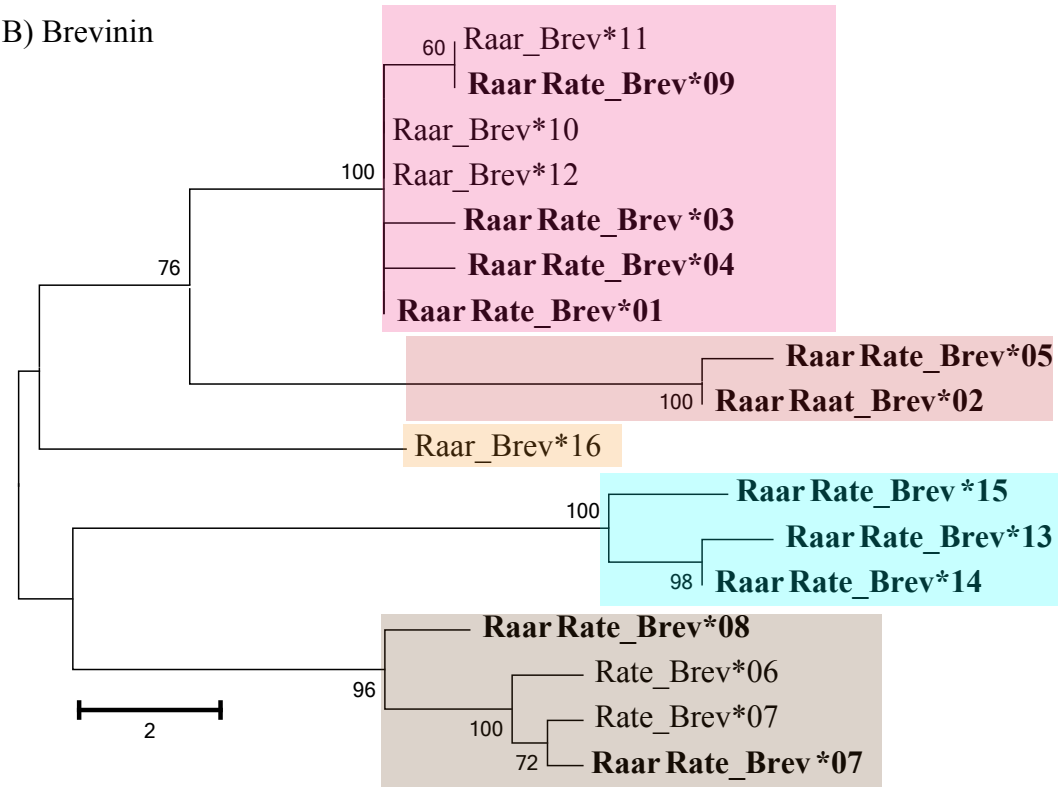

C) Palustrin

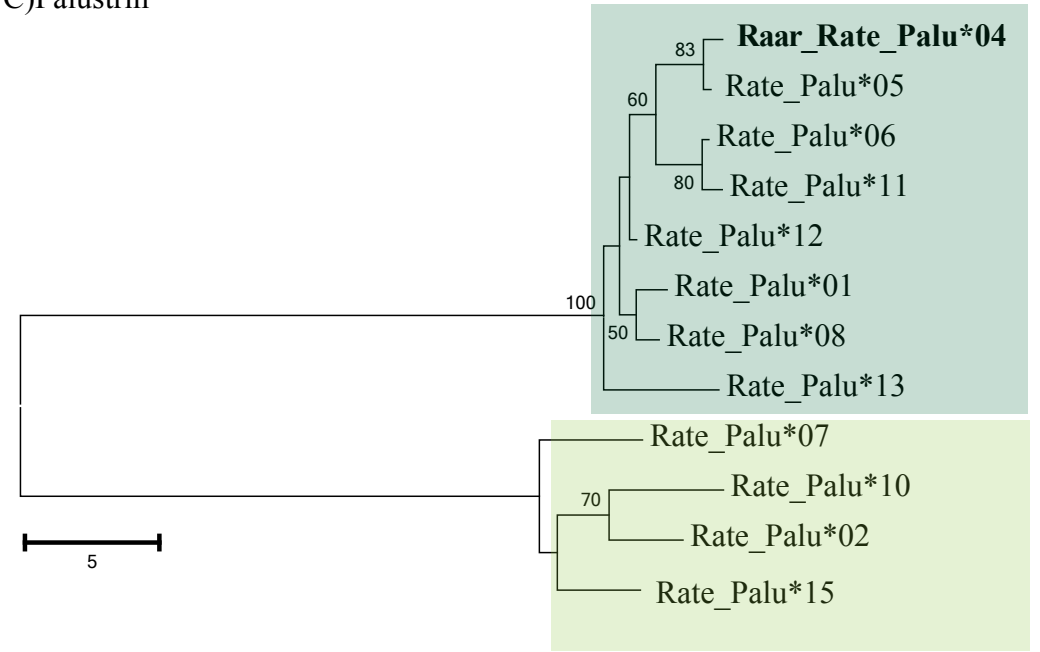

Supplement: Supplementary file 7 — Additional file 7: Figure 5. Molecular phylogram of nucleotide sequences of ranid antimicrobial peptides reconstructed with neighbor joining method for: a) Temporin, b) Brevinin and c) Palustrin. Bootstrap values from 1000 replicates greater than 50% are indicated on branches. Alleles that belong to the same group are included in the same colored square. Alleles shared by both R. arvalis and R. temporaria are shown in bold. Valid alleles were named following the nomenclature by Klein (1975) for MHC loci: a four-digit abbreviation of the species name followed by species_gene*numeration, e.g. Raar_Brev*01. [file 12863_2020_839_MOESM7_ESM.pdf]

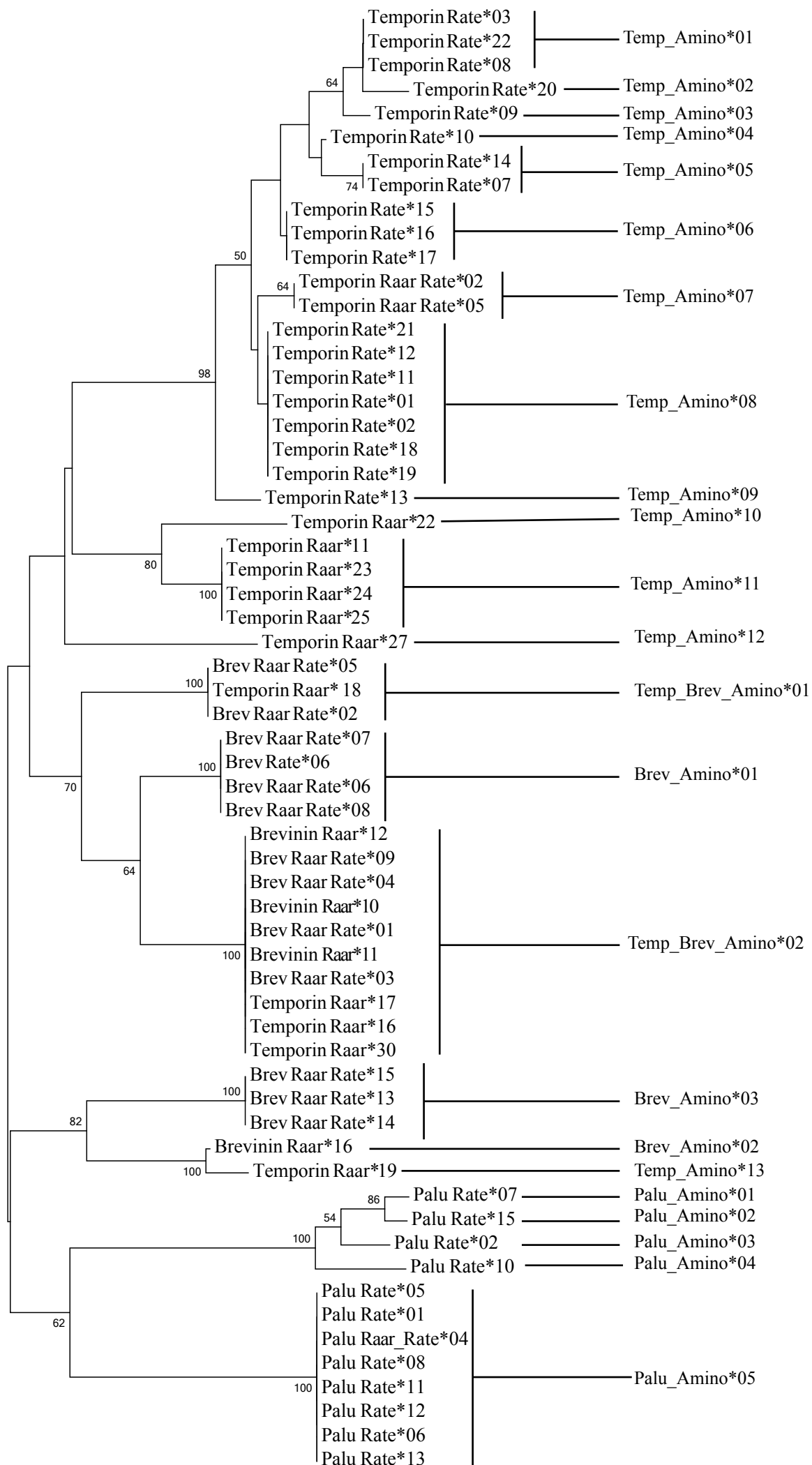

Supplement: Supplementary file 9 — Additional file 9: Figure 7. Molecular phylogram based on the amino-acid sequences was reconstructed with neighbor join methods. Name of the valid allelic variant were named following the nomenclature suggested by Klein (1975) for MHC loci: a four-digit abbreviation of the species name followed by spe-cies_gene*numeration, e.g. Raar_Brev*01. Nucleotide sequences function-ally indentical were named by gene_Amino*numeration. Bootstrap values from 1000 replicates greater than 50% are indicated on branches. [file 12863_2020_839_MOESM9_ESM.pdf]

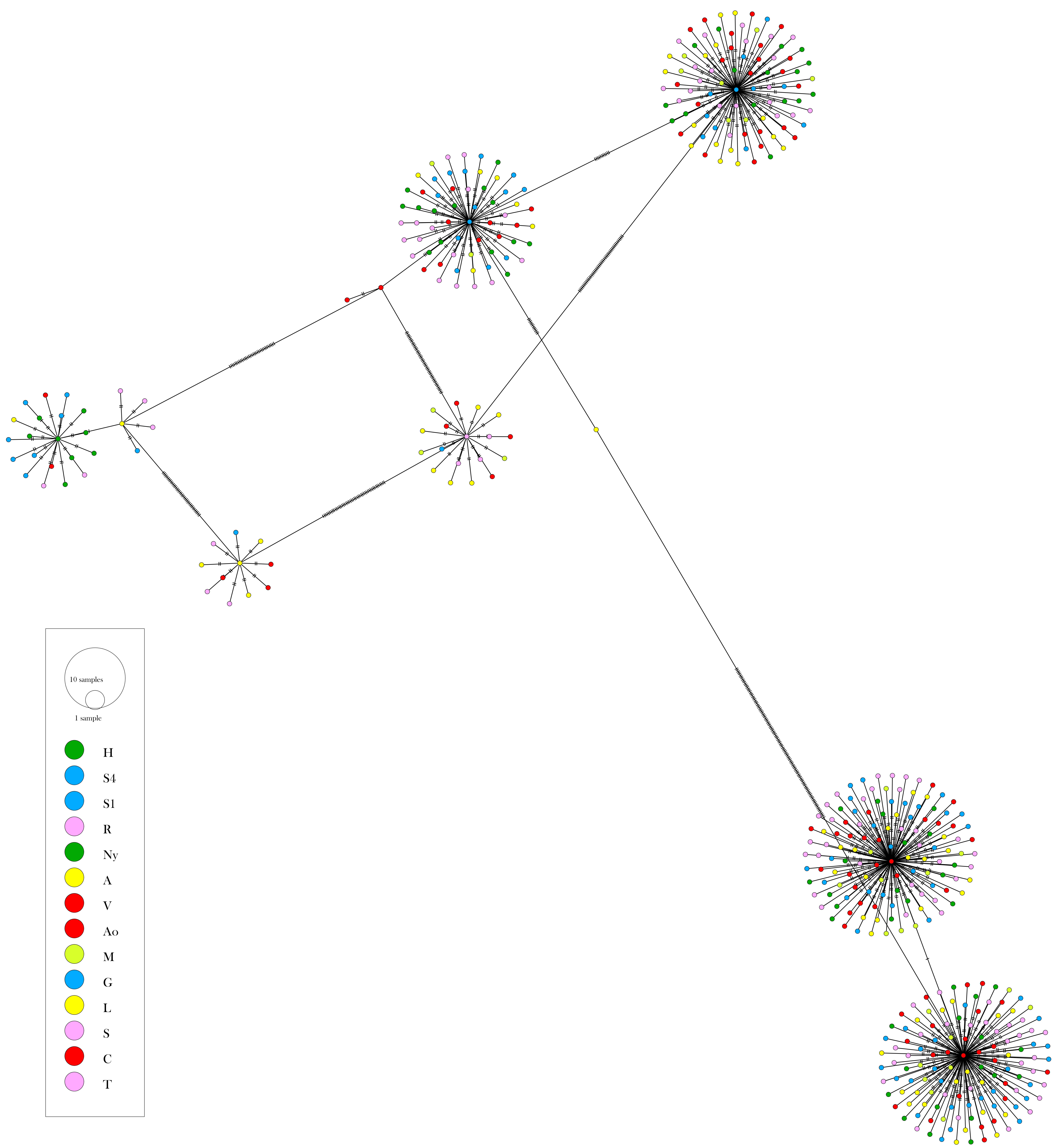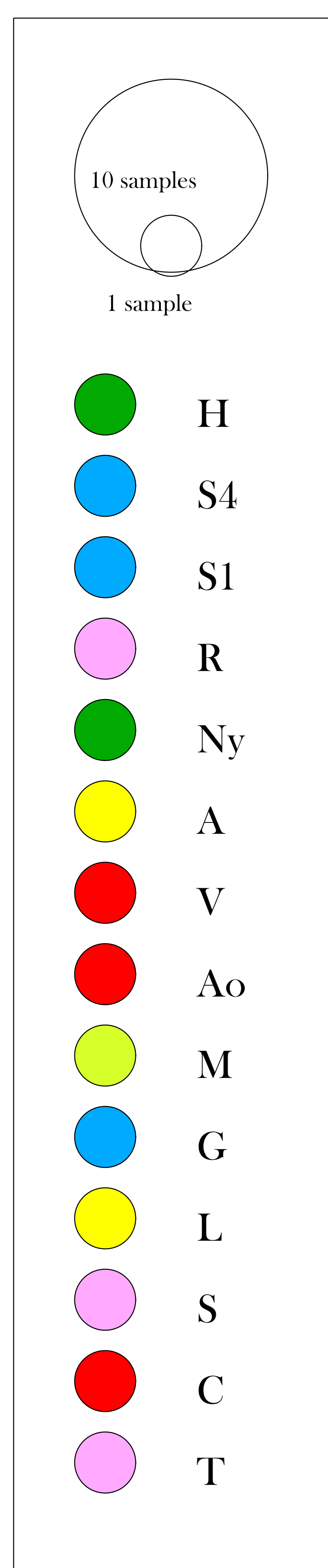

Supplement: Supplementary file 10 — Additional file 10: Figure 8. Minimum Spanning Network representing Temporin nucleotide variants in R.arvalis. Every circle represents one single individual. Populations within regions along the gradient are represented with the same colour. [file 12863_2020_839_MOESM10_ESM.pdf]

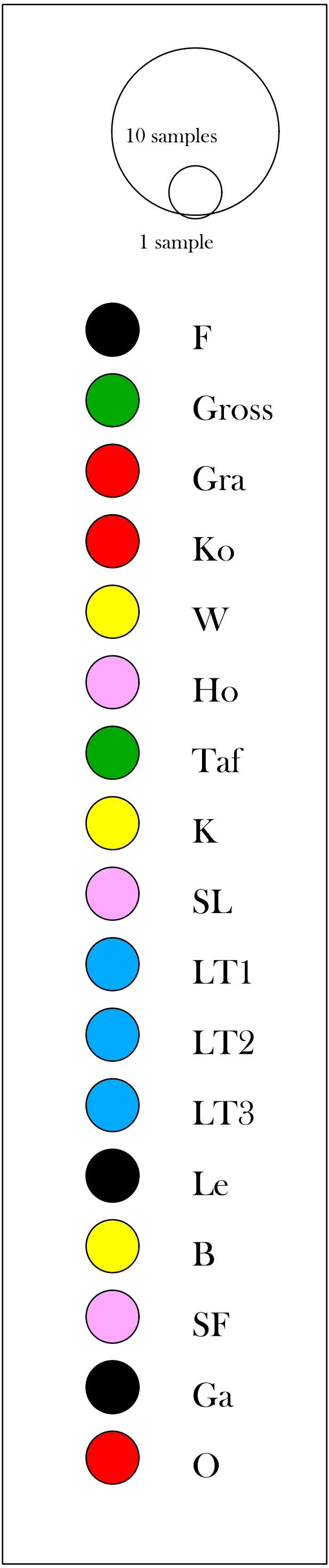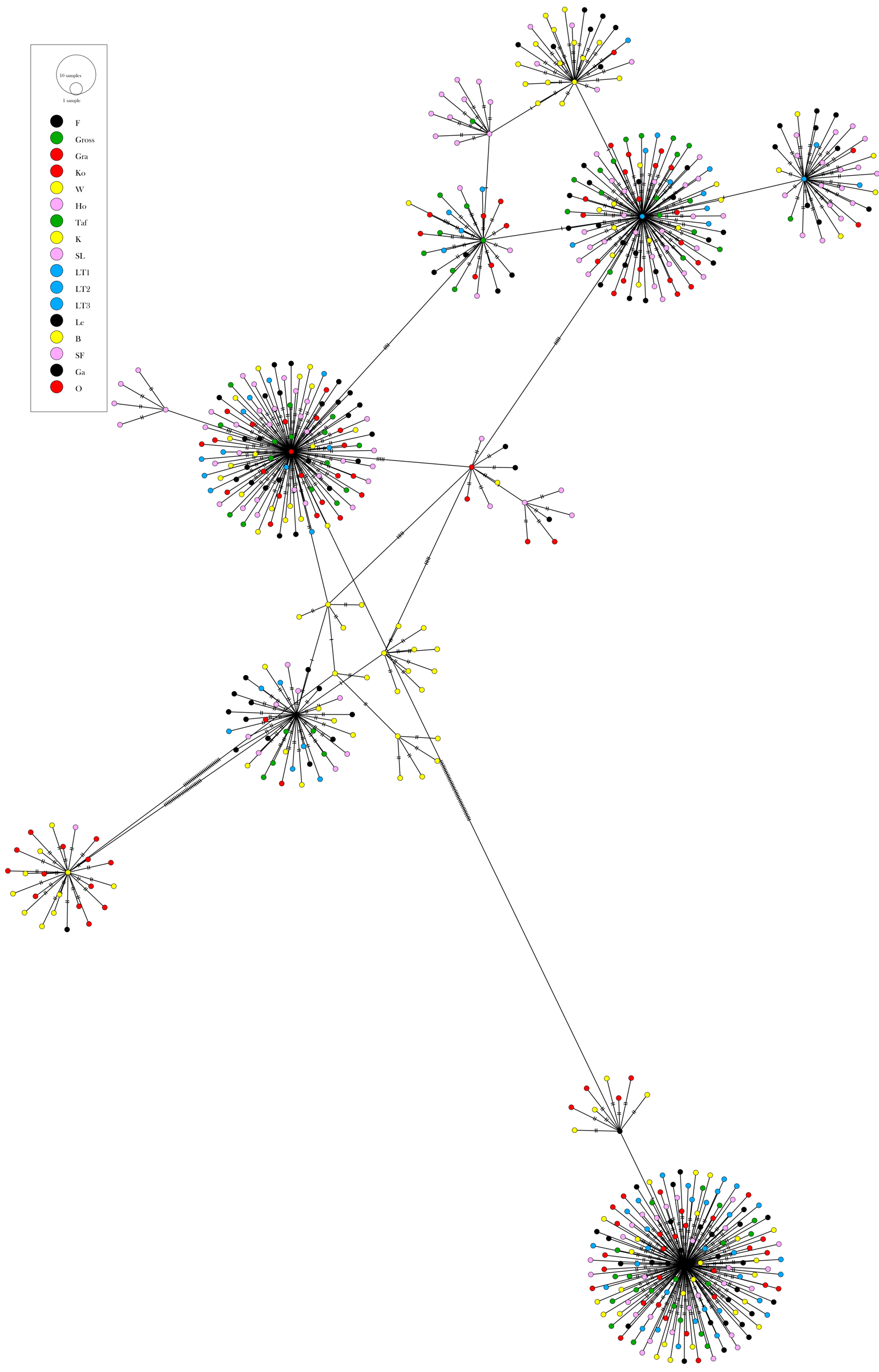

Supplement: Supplementary file 11 — Additional file 11: Figure 9. Minimum Spanning Network representing Temporin nucleotide variants in R.temporaria. Every circle represents one single indvidual. Populations within regions along the gradient are represented with the same colour. [file 12863_2020_839_MOESM11_ESM.pdf]

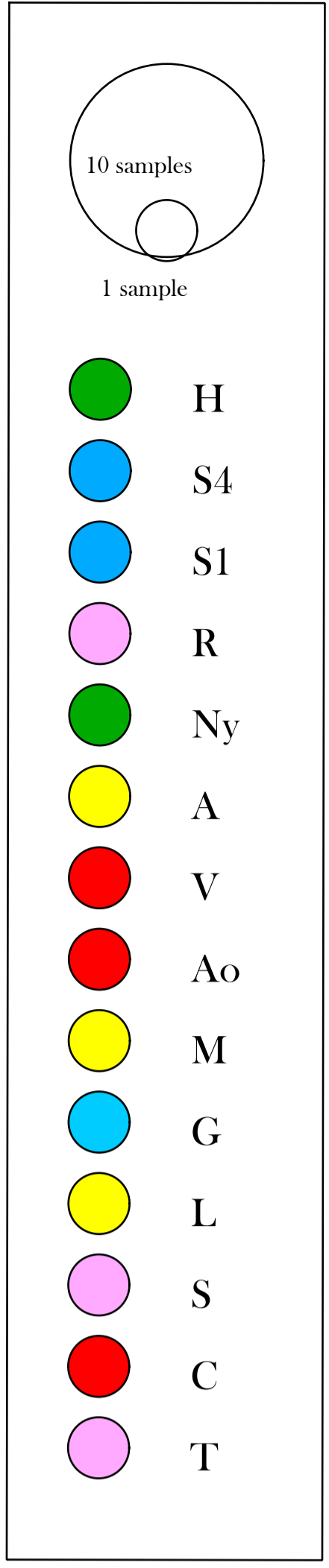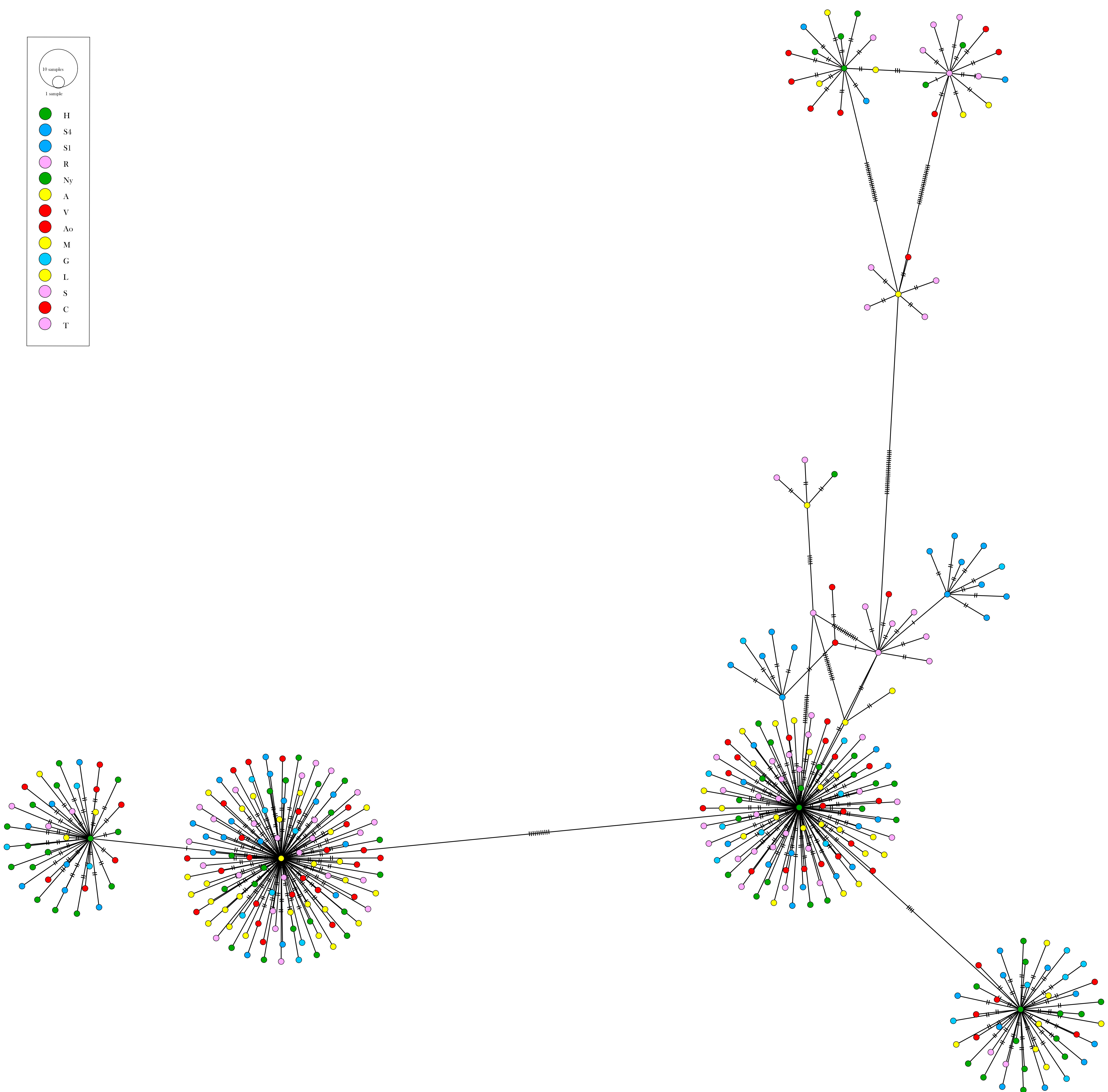

Supplement: Supplementary file 12 — Additional file 12: Figure 10. Minimum Spanning Network representing Brevinin nucleotide variants in R.arvalis. Every circle represents one single indvidual. Populations within regions along the gradient are represented with the same colour. [file 12863_2020_839_MOESM12_ESM.pdf]

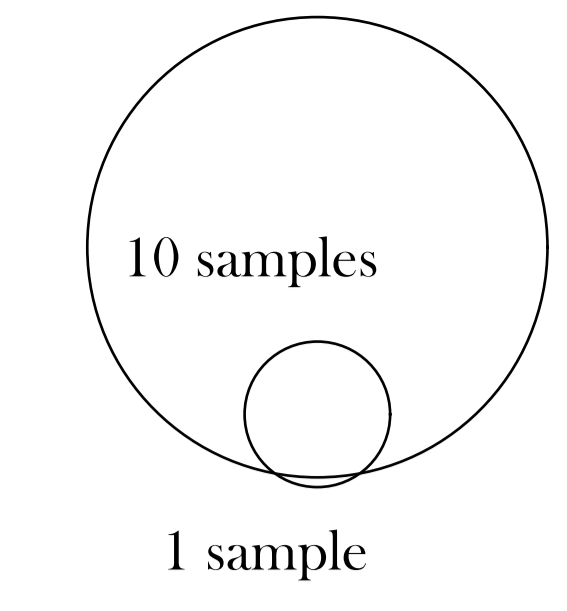

- F
- Gross
- Gra
- Ko
- W
- Ho
- Taf
- K
- SL
- LT1
- LT2
- LT3
- Le
- B
- SF
- Ga
- O

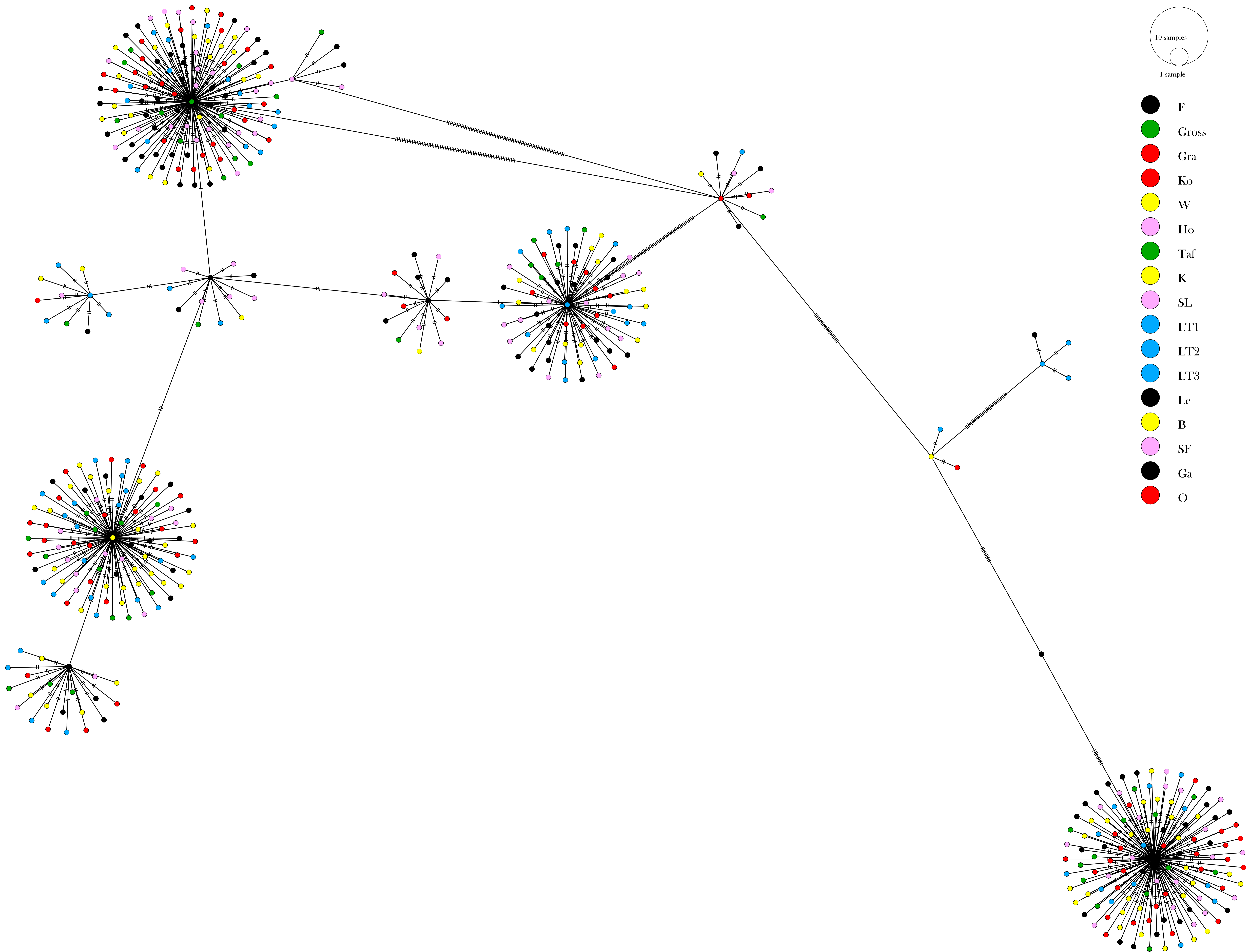

Supplement: Supplementary file 13 — Additional file 13: Figure 11. Minimum Spanning Network representing Brevinin nucleotide variants in R. temporaria. Every circle represents one single indvidual. Populations within regions along the gradient are represented with the same colour. [file 12863_2020_839_MOESM13_ESM.pdf]

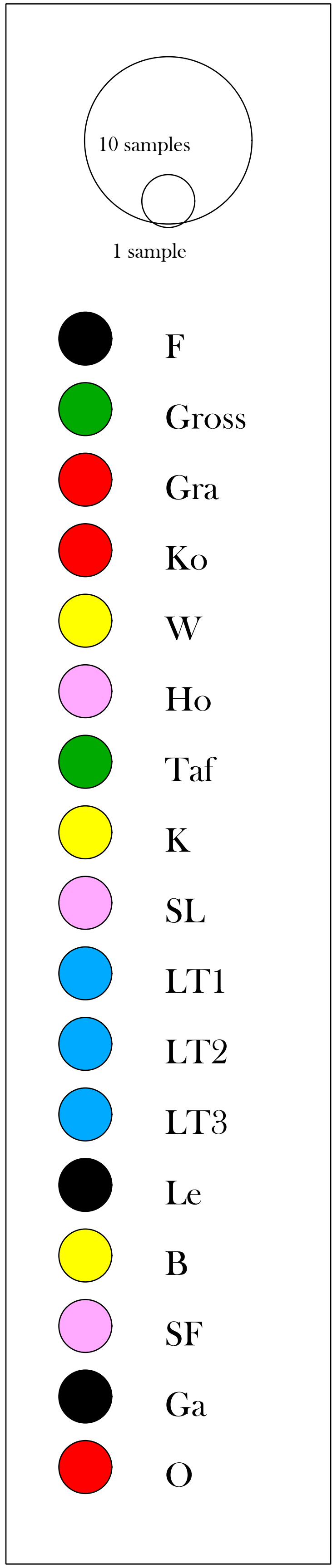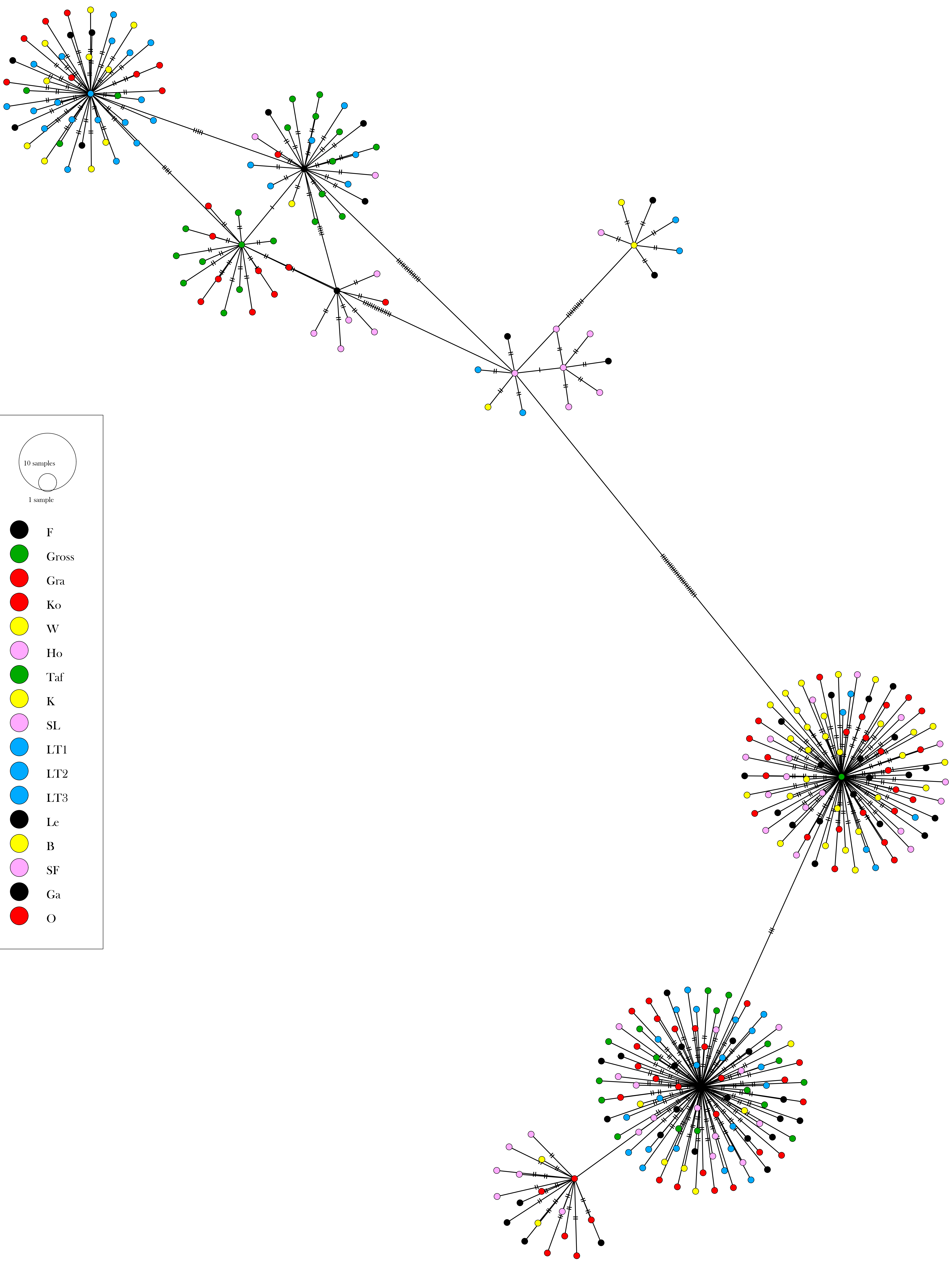

Supplement: Supplementary file 14 — Additional file 14: Figure 12. Minimum Spanning Network representing Palustrin nucleotide variants in R. temporaria. Every circle represents one single indvidual. Popu-lations within regions along the gradient are represented with the same colour. [file 12863_2020_839_MOESM14_ESM.pdf]

A)

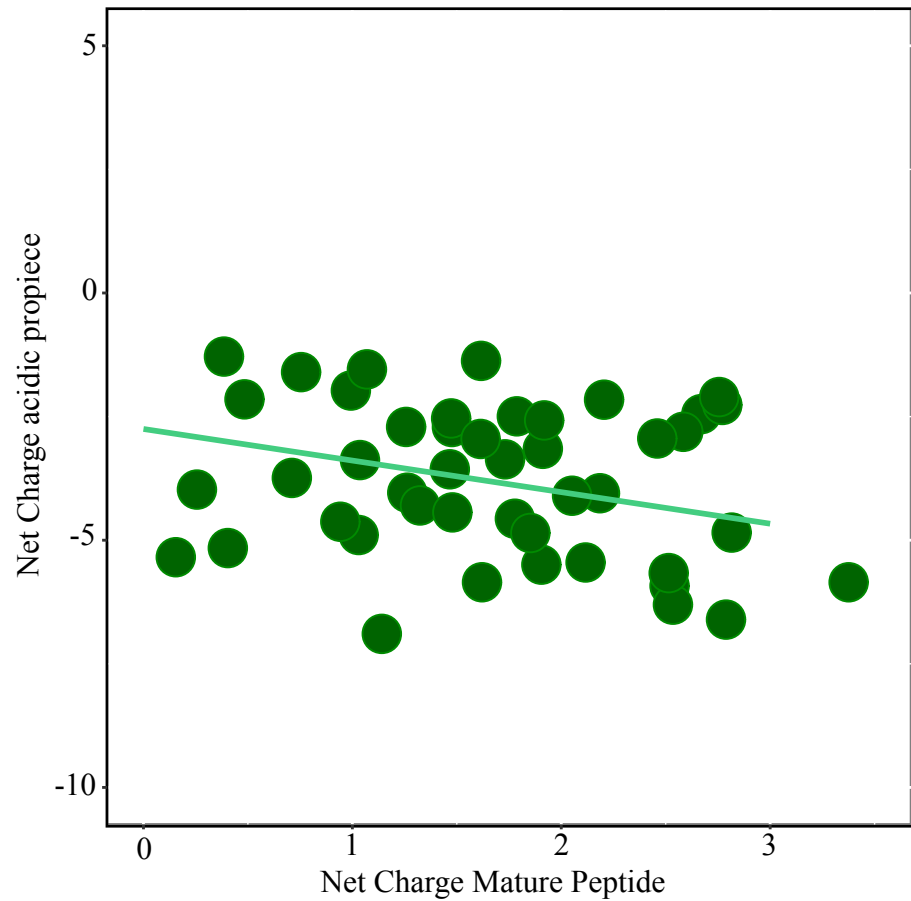

B)

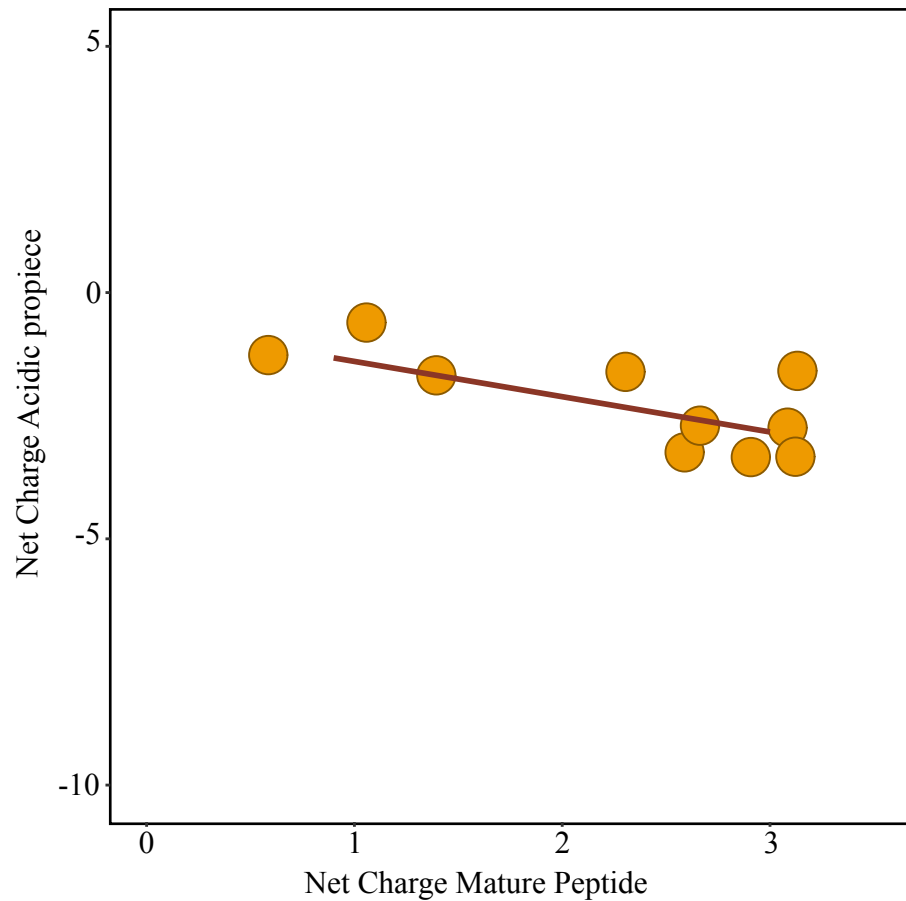

Supplement: Supplementary file 15 — Additional file 15: Figure 13. Net charge of the Acidic Propiece domain is relatively related to the net charge of the Mature Peptide domain for a) Temporin and Brevinin sequences and b) Palustrin sequences. The regression line is plotted in green and brown, respectively in A) and B). [file 12863_2020_839_MOESM15_ESM.pdf]

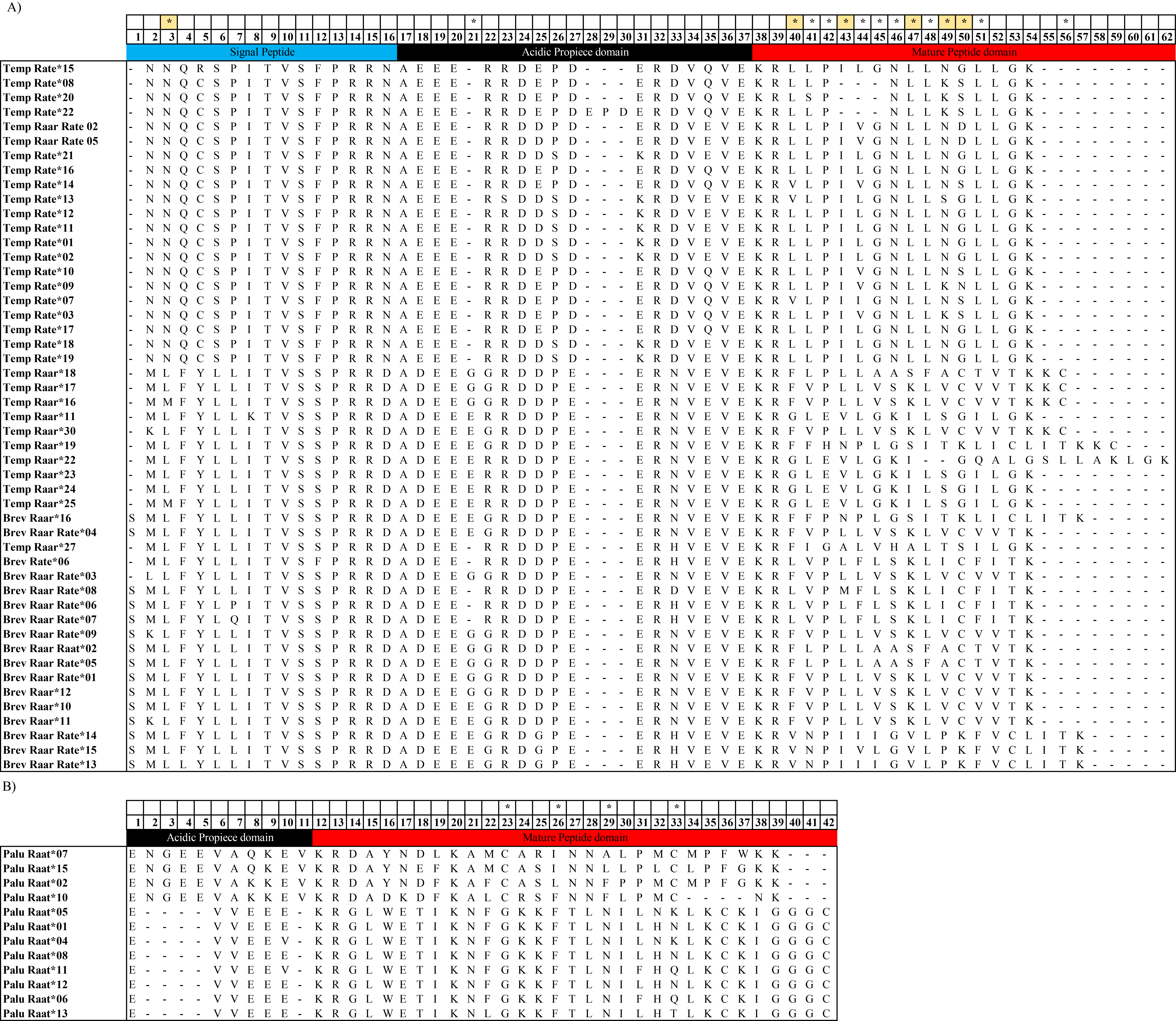

Supplement: Supplementary file 16 — Additional file 16: Figure 14. Antimicrobial peptide alignment. The Signal Peptide is rep-resented in blue, the Acidic Propiece in black and the Mature Peptide in red. Codon under selection were marked with an asterisk (*). Codons under selection detected at least by two different methods are colored in yellow. A) represent Temporin-Brevinin group of genes and B) palustrin group of genes, respectively. [file 12863_2020_839_MOESM16_ESM.png]
