# Supplementary material for: Antimicrobial peptide and sequence variation along a latitudinal gradient in two anurans
Source: BMC Genet. 2020 Mar 30;21:38. doi: 10.1186/s12863-020-00839-1 (PMC7106915; doi:10.1186/s12863-020-00839-1)
Supplement: Supplementary file 5 — Additional file 5: Figure 3. Colour scheme for the allele frequency pie charts for nucleotide sequences represented in Additional file 4: Figure 2. [file 12863_2020_839_MOESM5_ESM.pdf]

Temporin *R.arvalis*

- Raar_Temp*11
- Raar_Temp*16
- Raar_Temp*17
- Raar_Temp*18
- Raar_Temp*19
- Raar_Temp*22
- Raar_Temp*23
- Raar_Temp*24
- Raar_Temp*25
- Raar_Temp*27
- Raar_Temp*30
- Raar_Rate_Temp*02
- Raar_Rate_Temp*05

Brevinin *R.arvalis*

- Raar_Brev*10
- Raar_Brev*11
- Raar_Brev*12
- Raar_Brev*16
- Raar_Rate_Brev*01
- Raar_Rate_Brev*02
- Raar_Rate_Brev*03
- Raar_Rate_Brev*04
- Raar_Rate_Brev*05
- Raar_Rate_Brev*07
- Raar_Rate_Brev*08
- Raar_Rate_Brev*09
- Raar_Rate_Brev*13
- Raar_Rate_Brev*14
- Raar_Rate_Brev*15

Palustrin *R.arvalis*

- Raar_Rate_Palu*04

Temporin *R. Temporaria*

- Raar_Rate_temp*02
- Raar_Rate_Temp*05
- Rate_temp*01
- Rate_Temp*02
- Rate_Temp*03
- Rate_Temp*07
- Rate_temp*08
- Rate_Temp*09
- Rate_Temp*10
- Rate_Temp*11
- Rate_Temp*12
- Rate_Temp*13
- Rate_Temp*14
- Rate_Temp*15
- Rate_Temp*16
- Rate_Temp*17
- Rate_Temp*18
- Rate_Temp*19
- Rate_Temp*20
- Rate_Temp*21
- Rate_Temp*22

Brevinin *R.temporaria*

- Rate_Brev*06
- Raar_Rate_Brev*01
- Raar_Rate_Brev*02
- Raar_Rate_Brev*03
- Raar_Rate_Brev*04
- Raar_Rate_Brev*05
- Rate_Brev*07
- Raar_Rate_Brev*07
- Raar_Rate_Brev*08
- Raar_Rate_Brev*09
- Raar_Rate_Brev*13
- Raar_Rate_Brev*14
- Raar_Rate_Brev*15

Palustrin *R.temporaria*

- Rate_Palu*01
- Rate_Palu*02
- Raar_Rate_Palu*04
- Rate_Palu*05
- Rate_Palu*06
- Rate_Palu*07
- Rate_Palu*08
- Rate_Palu*10
- Rate_Palu*11
- Rate_Palu*12
- Rate_Palu*13
- Rate_Palu*15
