# Supplementary material for: Antimicrobial peptide and sequence variation along a latitudinal gradient in two anurans
Source: BMC Genet. 2020 Mar 30;21:38. doi: 10.1186/s12863-020-00839-1 (PMC7106915; doi:10.1186/s12863-020-00839-1)
Supplement: Supplementary file 8 — Additional file 8: Figure 6. Colour scheme for the amino-acid frequency pie charts represented in Additional file 9: Figure 7. [file 12863_2020_839_MOESM8_ESM.pdf]

Temporin Aminoacid R.arvalis

- **Temp_Amino*07**
- Temp_Amino*10
- Temp_Amino*11
- Temp_Amino*13
- **Temp_Brev_Amino*01**
- **Temp_Brev_Amino*02**

Brevinin Aminoacids R.arvalis

- **Brev_Amino*01**
- Brev_Amino*02
- **Brev_Amino*03**
- **Temp_Brev_Amino*01**
- **Temp_Brev_Amino*02**

Palustrin Aminoacid *R.arvalis*

- **Palu_Amino*05**

Temporin Aminoacid R.temporaria

- Temp_Amino*01
- Temp_Amino*02
- Temp_Amino*03
- Temp_Amino*04
- Temp_Amino*05
- Temp_Amino*06
- **Temp_Amino*07**
- Temp_Amino*08
- Temp_Amino*09

Brevinin Aminoacid *R.temporaria*

- **Brev_Amino*01**
- **Brev_Amino*03**
- **Temp_Brev_Amino*01**
- **Temp_Brev_Amino*02**

Palustrin Aminoacid *R.temporaria*

- Palu_Amino*01
- Palu_Amino*02
- Palu_Amino*03
- Palu_Amino*04
- **Palu_Amino*05**
